# Intermolecular electron transfer in radical SAM enzymes as a new paradigm for reductive activation

**DOI:** 10.1016/j.jbc.2023.105058

**Published:** 2023-07-17

**Authors:** Karsten A.S. Eastman, Andrew S. Jochimsen, Vahe Bandarian

**Affiliations:** University of Utah, Department of Chemistry, Salt Lake City, Utah, USA

**Keywords:** *S*-adenosyl-L-methionine (SAM), electron transfer, enzymatic activation, enzyme mechanism, iron–sulfur protein, radical SAM

## Abstract

Radical S-adenosyl-L-methionine (rSAM) enzymes bind one or more Fe-S clusters and catalyze transformations that produce complex and structurally diverse natural products. One of the clusters, a 4Fe-4S cluster, binds and reductively cleaves SAM to generate the 5′-deoxyadenosyl radical, which initiates the catalytic cycle by H-atom transfer from the substrate. The role(s) of the additional auxiliary Fe-S clusters (ACs) remains largely enigmatic. The rSAM enzyme PapB catalyzes the formation of thioether cross-links between the β-carbon of an Asp and a Cys thiolate found in the PapA peptide. One of the two ACs in the protein binds to the substrate thiol where, upon formation of a thioether bond, one reducing equivalent is returned to the protein. However, for the next catalytic cycle to occur, the protein must undergo an electronic state isomerization, returning the electron to the SAM-binding cluster. Using a series of iron–sulfur cluster deletion mutants, our data support a model whereby the isomerization is an obligatorily intermolecular electron transfer event that can be mediated by redox active proteins or small molecules, likely *via* the second AC in PapB. Surprisingly, a mixture of FMN and NADPH is sufficient to support both the reductive and the isomerization steps. These findings lead to a new paradigm involving intermolecular electron transfer steps in the activation of rSAM enzymes that require multiple iron–sulfur clusters for turnover. The implications of these results for the biological activation of rSAM enzymes are discussed.

Radical S-adenosyl-L-methionine (rSAM) enzymes comprise a superfamily of metalloenzymes that catalyze a wide range of radical-mediated transformations (1). All rSAM enzymes bind a 4Fe-4S cubane cluster (RS cluster) with three Fe atoms coordinating three Cys thiolate that are nearly always located within a conserved CxxxCxxC motif (2, 3, 4). The fourth Fe coordinates the α-amino and α-carboxylate moieties of the SAM cofactor (5, 6, 7). While the cluster can exist in either the +2 or +1 oxidation states, only the reduced cluster catalyzes the reductive cleavage of SAM to form a 5′-deoxyadenosyl radical (dAdo⋅), which initiates the catalytic cycle of most rSAM enzymes *via* H-atom transfer from the substrate (8).

Several classes of rSAM enzymes are recognized (9). Class 1 enzymes use SAM catalytically, returning the SAM-binding [4Fe-4S] cluster to the +1 state. Class 2 enzymes generate a glycyl radical on a protein substrate. Most rSAM enzymes belong to class 3, which use SAM stoichiometrically. While the RS cluster is housed within a partial TIM barrel, most characterized rSAM enzymes contain additional Fe-S clusters, referred to as auxiliary clusters (ACs), that are housed in C-terminal extensions of the TIM barrel domain (10, 11, 12, 13). The SPASM-(subtilosin maturase, PQQ maturase, anaerobic sulfatase maturation enzyme, and mycofactocin maturase (14, 15, 16)) domain proteins contain two ACs, while Twitch-domain proteins house one AC (17). The best characterized RS enzymes containing these AC motifs are those that insert sulfur into their substrate, such as lipoic acid synthase and biotin synthase (18, 19, 20, 21, 22). In these systems, the AC that is proximal to the RS cluster is the source of the sulfur that is inserted into the substrate. With a few exceptions (23, 24, 25, 26, 27, 28, 29), the role of the analogous ACs in other rSAM enzymes remain elusive. In the case of SPASM domain–containing rSAM enzymes, the role of the distal cluster remains enigmatic.

The catalytic cycle of all rSAM enzymes requires the reduction of the RS cluster from a catalytically inactive +2 state to the catalytically active +1 state. This reduction is usually mimicked *in vitro* using strong chemical reductants such as sodium dithionite (NaDT) or titanium citrate (3, 27, 30). Reichard and colleagues were the first to show that flavodoxin (FldA) and flavodoxin reductase (FPR) can also support turnover by relaying electrons from NADPH (31). This latter discovery was initially made with the *Escherichia coli* homologs of the two proteins and has been adopted by most laboratories working on rSAM enzymes. Ironically, this approach is often referred to as using the biologically or physiologically relevant reducing system to activate rSAM enzymes (3, 30, 32, 33, 34). Although other homologs of FldA and FPR have been used successfully with rSAM enzymes (30), the pair from *E. coli* has demonstrated activation of rSAM enzymes from nearly every kingdom of life. However, considering the diverse surface topologies of many radical rSAM enzymes (35), it seems unlikely that the reducing pair found in *E. coli* is a universal biological reducing system for all rSAM enzymes, with its successful utilization to date representing exceptions. Indeed, a flavoprotein such as FldA is not the only possible candidate for reducing system *in vivo.* For example, Booker and colleagues have shown that MiaB from *Thermatoga maritima* can be activated by an iron-sulfur-containing ferredoxin homolog from the same organism, where the activating electron is ultimately derived from NADPH *via* a ferredoxin-NADP^+^ oxidoreductase also from *T. maritima* (36). An interesting recent example of a reducing system for rSAM enzymes is a modified yellow fluorescent protein analogue, PSP2, with a photoactivatable prosthetic group. When photoreduced, PSP2 supports turnover of BtrN and Dph2; presumably the organic prosthetic group is the electron donor (37). Therefore, the key requirement for inactivation of rSAM proteins appears to be the presence of a species that can catalyze one-electron reduction. These observations highlight a crucial gap in understanding how rSAM enzymes are activated *in vivo* (32).

PapB (WP_019688962.1) is a class 3, SPASM-domain rSAM enzyme that catalyzes the formation of thioether cross-links between Cys and Asp residues in a ribosomally encoded and posttranslationally modified polypeptide (RiPP), PapA (38, 39). The genes that encode for PapA and PapB, originating from *Paenibacillus polymyxa*, are located in adjacent open reading frames (*orfs*). Previous studies of this system have shown six CX_3_D motifs in the PapA substrate that are cross-linked by PapB catalysis. However, PapB is active with a peptide substrate that has only one of these CX_3_D sites (minimal substrate PapA, msPapA), thereby simplifying the biochemical studies of the protein (38). PapB is among the most active rSAM enzymes and has a robust turnover number of 7.4 s^−1^ (40). While the structure of PapB remains unknown, a Robetta-predicted structure is shown in Figure 1*A* (41). Sequence comparisons with other homologs and inductively coupled plasma mass spectrometry (ICP-MS) measurement of the Fe content suggest that PapB is a SPASM-domain protein and can bind three [4Fe-4S] clusters (38, 40). The RS cluster activates SAM toward reductive cleavage to form dAdo⋅ (Fig. 1*B*), which abstracts a H-atom from the C3 of the Asp residue to generate a substrate radical (Fig. 1*C*). Extended X-ray absorption fine-structure studies have shown one of the two ACs is responsible for binding and activating Cys thiol of the CX_3_D motif (24). The formation of the thioether cross-link that oxidizes the C3 of Asp and generates a single electron is followed by disengagement of the sulfur from the cluster. It has been proposed that this electron is stored in AC1 (24). Currently, the role of the third cluster, AC2, is unknown, but it is possible that AC1 and AC2 may collectively be involved in the reactivation of the RS cluster. As with many other rSAM enzymes where a reducing equivalent remains in the active site of the enzyme at the end of the catalytic cycle, it is thought that the electron will return to the RS cluster, thereby reactivating the enzyme for the next round of catalysis (42). Indeed, in several systems where it has been examined, prereduction of the enzyme is sufficient to permit multiple turnovers to occur without the addition of exogenous reducing equivalents (43, 44, 45). In general, the reactivation of these prereduced systems is thought to occur by electron transfer back to the RS cluster, although there is no direct evidence for whether the transfer is *intra-* or *inter*molecular.

**Figure 1:**
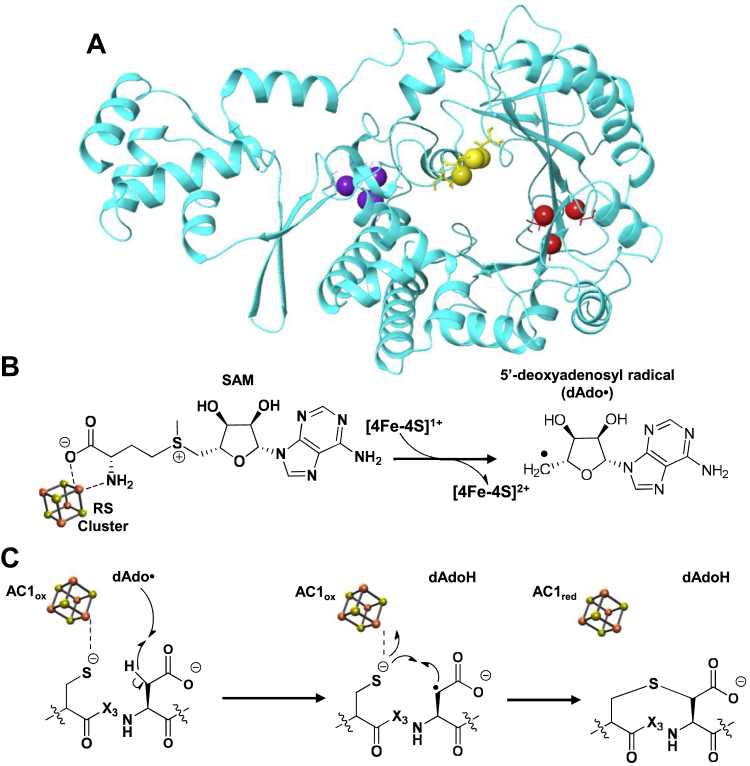
**Robetta-predicted** (41) **structural model of PapB and the thioether formation mechanism.***A*, the conserved Cys residues in the sequence alignment (Fig. S2) are predicted to occur in three distinct regions. The Cys residues that ligate the three 4Fe-4S clusters in PapB are shown in red (RS), yellow (AC1), and purple (AC2). *B*, upon reduction of the RS cluster, the homolytic cleavage of SAM produces a dAdo⋅. *C*, the dAdo⋅ abstracts an H-atom from the position α to the carboxylate in the peptide, generating a substrate radical. Thioether bond formation is coupled to disengagement from AC1 (24).

The true biological mechanism behind the two reduction steps necessary for the activation and post-catalysis reactivation of the RS cluster in PapB and other rSAM enzymes are enigmatic. Current hypotheses suggest that the first step may require flavodoxin or flavodoxin-like systems for the reductive activation *in vivo* (35). However, the Booker study discussed above suggests that one cannot eliminate the possibility that *in vivo* reduction may not require a dedicated protein or class of enzymes (36). The second elusive step is the postcatalysis reactivation in rSAM enzymes that house multiple clusters, where the catalytic cycle returns the reducing equivalent back to the RS cluster to rearm it. Intramolecular electron transfer has often been invoked for this step, however, without evidence.

This study explores the reductive activation of PapB and the fate of the electron that remains in the protein at the end of the catalytic cycle. In contrast to current models, our findings suggest that *intermolecular* electron transfer is necessary for each cycle of PapB to return the reducing equivalent back to the RS cluster to ready the system for another cycle. The studies described below include variants of PapB where each of the three clusters is deleted to provide evidence that the intermolecular electron transfer requires AC2. Our results support a new paradigm for the cellular activation of rSAM enzymes, as they show no specific activator is necessary. We demonstrate that, under anaerobic conditions, NADPH can reduce flavin mononucleotide (FMN), which in turn activates PapB. As proof of concept for intermolecular electron transfer, we present evidence for the transfer between two completely unrelated rSAM enzymes, one that is prereduced and the other oxidized. Under this scenario, we see reciprocal reductive activation of both systems when assayed *in situ*. These results suggest that the true biological reduction of rSAM may occur in more ways than considered previously as it does not require a specific biological reducing partner, but instead, it only needs one that can deliver the reducing equivalent in one-electron steps. The implications of these findings will be discussed in the context of a generalized model for the *in vivo* reductive activation of rSAM enzymes.

## Results

### Purification of PapB

PapB (∼55 kDa) was purified to homogeneity from an overexpressing *E. coli* strain (Fig. S1) and reconstituted with iron and sulfide as described (40). Amino acid and ICP-MS analysis of enzyme purified from three separate rounds of expression revealed that the protein contains 13.5 ± 0.3 mol of iron per mol of PapB (40). Sequence alignments with CteB (PDB:5WGG) (46) and anSME (PDB:4K37) (34) show that PapB conserves the 10 Cys residues that bind RS, AC1, and AC2 in CteB and anSME (Fig. S2). In the Robetta analysis (41) of PapB, these Cys residues are grouped in such manner as to support binding of the RS cluster (red), AC1 (yellow), and AC2 (purple) (Fig. 1). The iron content of reconstituted PapB used in the studies described below is sufficient to support formation of three [4Fe-4S] clusters.

### PapB is active in the absence of FPR

**Figure 2:**
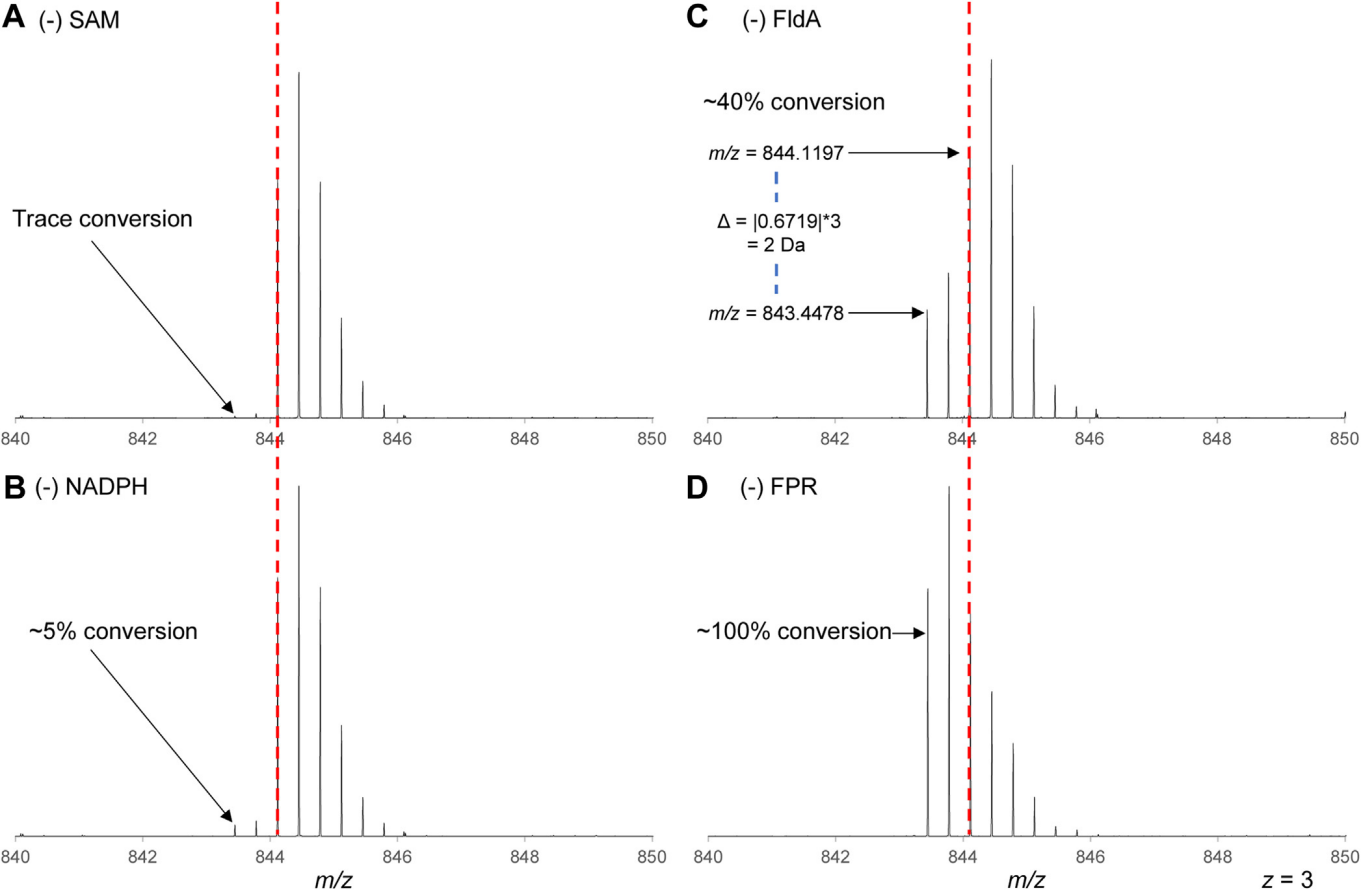
**Dependence of the activity of PapB on components of the reducing system.** PapB is fully active in the buffer solutions that contain FldA, FPR, SAM, and NADPH (see Fig. S3), with quantitative cross-linking of msPapA observed in 15 min. In a charge state of +3, a cross-link corresponds to a shift of −0.6719, which is a Δ2 Da (*m/z* = 843.4478, M = 2530.3434). *A*, control reactions with no SAM or (*B*) NADPH show only trace quantities of cross-linked peptide. *C*, by contrast, ∼40% completion is observed in the absence of FldA. *D*, nearly quantitative conversion to the cross-linked species is observed in the absence of FPR. The red dotted line signifies the unmodified monoisotopic mass of the msPapA peptide (*m/z* = 844.1197, M = 2532.3591).

The catalytic activity of PapB was readily assessed by analyzing the msPapA peptide by LC-MS for cross-linking after incubating with PapB, SAM, and either the reducing system of 2 mM NaDT or 25 μM FldA, 2 μM FPR, and 2 mM NADPH. It should be noted that all the assay mixtures also contained 2 mM dithiothreitol (DTT) to minimize the dimerization of the peptide by the formation of disulfides. A representative mass spectrometry (MS) (in positive ion mode) of the peptide in the presence and absence of full assay components is shown in Fig. S3. Figure 2 shows the *z* = 3 charge state of the msPapA peptide after incubation with PapB for 15 min under various conditions. The thioether modification led to a Δ2 amu in the *m/z* of the monoisotopic peak. Throughout this study, the extent of cross-linking is determined by comparisons of the intensity of the monoisotopic peak of the cross-linked species to the peak in the envelope that has contributions from the monoisotopic peak of the unmodified peptide (see Fig. S4 for simulated envelopes corresponding to 0%-100% turnover). In the presence of NADPH, FPR, FldA, and SAM, the msPapA substrate is quantitatively converted to the cross-linked species (Fig. S3). When SAM is excluded (Fig. 2*A*), a small amount of turnover is also observed, likely from the endogenous SAM from the *E. coli* expression host that copurifies with the protein. Furthermore, when NADPH is removed from the assay reaction (Fig. 2*B*), a small amount of cross-linked msPapA was observed. We attributed this to the presence of a small amount of prereduced PapB or FldA. Surprisingly, when either FldA (Fig. 2*C*) or FPR (Fig. 2*D*) was omitted from the reactions, significant amounts of cross-linked msPapA are observed. In the absence of FldA, ∼45% of the starting msPapA is transformed to product in 180 min, whereas in the absence of FPR, 100% conversion is observed.

The turnover observed in the absence of FPR is unexpected. To our knowledge, there is no evidence in the literature that FldA can be reduced by pyridine nucleotides. We posit that the activity results from either a small fraction of the purified FPR (or FldA) existing in the reduced form or their presence allows for the small amount of activity that is observed in the absence of NADPH (Fig. 2*B*) to become amplified. We cannot exclude the possibility that a small fraction of the PapB is purified in its reduced state and responsible for this background activity. It is also possible the DTT in these assays is reducing FldA or FPR, thereby furnishing a source of reducing equivalents for PapB; however, this reduction is slow (47) and in controls described below not significant in the 3-h timeframe of the experiment. Moreover, such a DTT-dependent reduction does not explain the substantial amount of additional turnover seen when comparing samples lacking NADPH with those that did not contain either FldA or FPR (compare Fig. 2*C* or Fig. 2, *B*–*D*). These latter observations point to a role for NADPH even in the absence of FdA or FPR.

### PapB activity with FMN and NADPH

**Figure 3:**
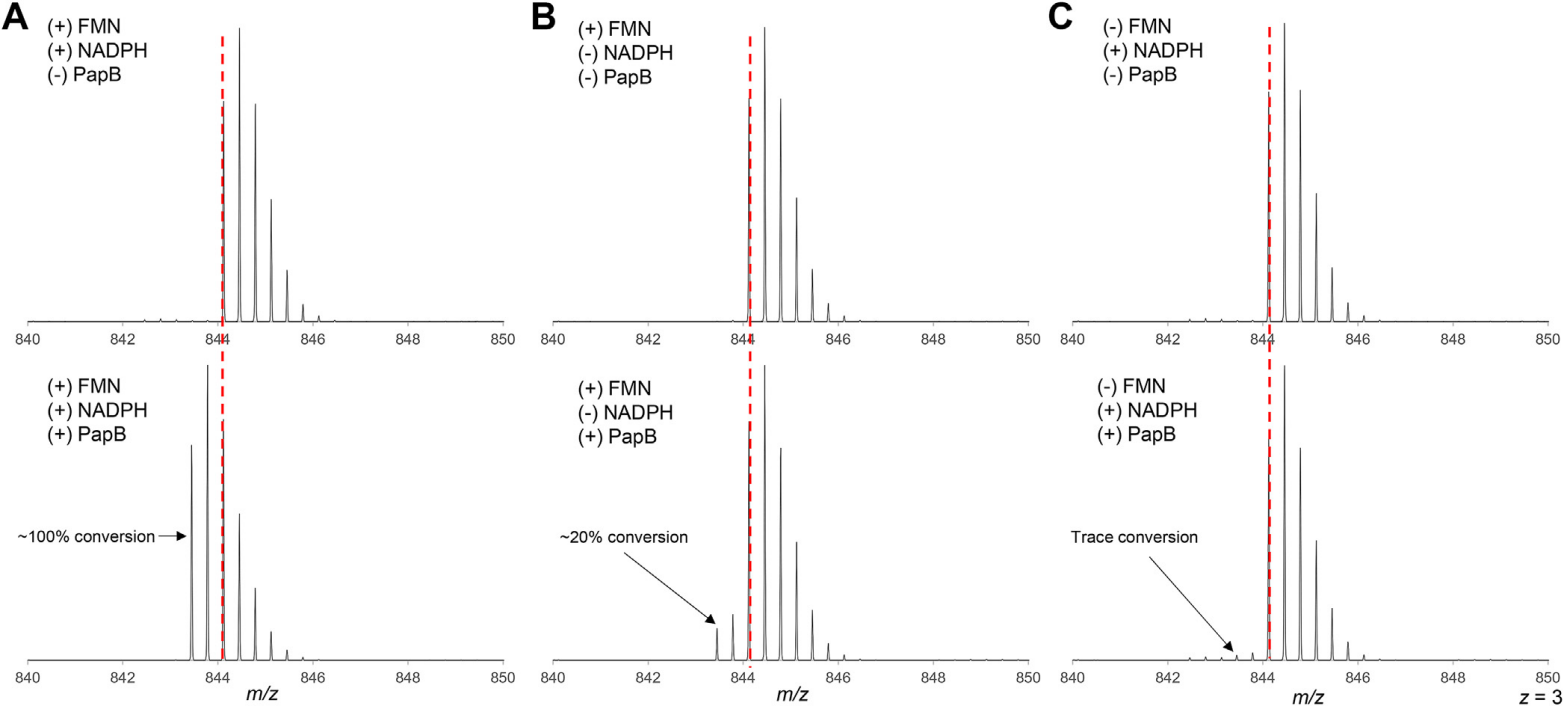
**PapB is active in the presence of FMN and NADPH.***A*, in the absence of PapB, no cross-linking is observed (*top row*). Full cross-linking of the peptide substrate is seen after 3 h when FMN and NADPH are present (*bottom row*). *B*, when NADPH is excluded, PapB still cross-links msPapA, albeit inefficiently. *C*, by contrast to (*A* and *B*) where FMN is present, removing FMN from the assay mixture leads to the formation of <1% cross-linked msPapA. The *red dashed lines* denote the calculated unmodified monoisotopic mass of msPapA (*m/z* = 844.1197).

Both FPR and FldA are flavoenzymes. To gain additional insights into the mechanism by which either FldA or FPR can support turnover, activity assays with PapB were carried out in the presence of FMN and NADPH (Fig. 3). Remarkably, in the presence of FMN and excess NADPH, we observe the quantitative conversion of msPapA to the cross-linked product (Fig. 3*A*). Alternatively, in the absence of NADPH, this activity is minimal, with only ∼30% of the substrate being cross-linked even after 180 min of incubation (Fig. 3*B*). As with the experiments shown in Figure 2, 2 mM DTT is included in these assays; therefore, the residual activity could result from reduction of PapB by DTT-reduced FMN. Interestingly, in the absence of FMN there is no measurable product formed (Fig. 3*C*), clearly establishing that the flavin cofactor is the likely conduit of electrons from NADPH to PapB. NADPH is an obligatory hydride donor, and therefore, it is not surprising that it is unable to support turnover of PapB in the absence of a one-electron donor.

### FMN or FldA can be reduced by NADPH

**Figure 4:**
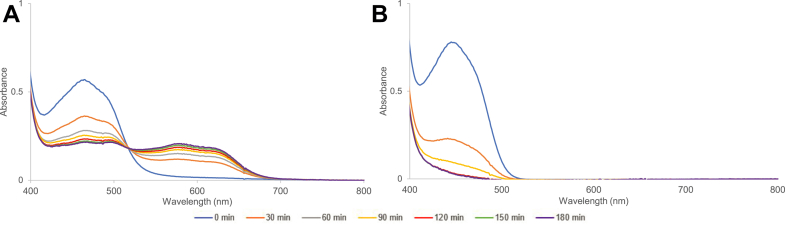
**FldA and FMN are reduced in the presence of NADPH.** A solution containing (*A*) 55 μM FldA or (*B*) FMN was incubated with 2 mM NADPH and spectra were obtained over a period of 180 min. While the flavin is reduced in both cases, the spectral changes in (*A*) suggest that FldA is reduced to the semiquinone, likely from equilibration of oxidized and two-electron reduced. FMN is reduced to the hydroquinone form. Each experiment was carried out in triplicate, but (*A* and *B*) show a representative example.

The above results implicate NADPH-reduced FldA (or FMN) in the activation of PapB. To assess this, we carried out assays in which 60 μM FldA or FMN was incubated with 2 mM NADPH in 50 mM Pipes·NaOH (pH 7.4) containing 300 mM KCl and the UV-visible spectra were monitored over 180 min (Fig. 4). Control experiments in the presence of 2 mM DTT in place of NADPH are shown in Fig. S5. In the presence of NADPH, FldA is reduced to the semiquinone species (Fig. 4*A*) and FMN to the hydroquinone (Fig. 4*B*). Within the 180-min timeframe, neither FldA (Fig. S5*A*) nor FMN (Fig. S5*B*) is reduced in the presence of 2 mM DTT (Fig. S5). To our knowledge, the ability of NADPH to directly reduce FMN or FldA has not been reported previously and may reflect the strictly anaerobic conditions employed in the assays; these results are highly significant as they allow for a new paradigm for the reductive activation of rSAM enzymes. The fact that PapB activity is observed when either FldA or FMN is present along with NADPH suggests that either the hydroquinone or semiquinone state of FMN can reduce the RS cluster of PapB to initiate catalysis (see Figs. 2 and 3). Our interpretation of the difference in the fate of flavin bound to FldA and free in solution is that, in both cases, the hydroquinone is the initial product, but with FldA, reduced FldA in the hydroquinone state can reduce oxidized FldA generating two equivalents of Fld with bound semiquinone.

In our interpretation of the data presented in Figures 2 and 3, we could not account fully for the PapB activity that is observed when NADPH was removed from assays containing FPR/FldA (Fig. 3*B*) or FldA (Fig. 2*B*). Considering that the control experiment shows the inability of 2 mM DTT alone to reduce FMN, even after 3 h (Fig. S5), the observed residual activities can be rationalized *only* if the oxidized flavin (either in FldA or FMN in solution) is playing a previously undetermined electron transfer role that is stimulatory.

### Oxidized FldA and FMN stimulate activity of prereduced PapB

**Figure 5:**
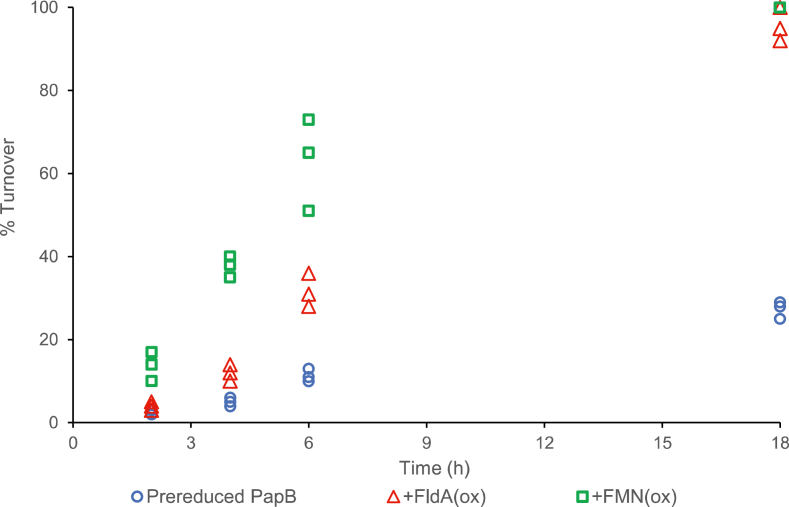
**Prereduced PapB can cross-link Y17W msPapA and is more efficient in the presence of either oxidized FldA or oxidized FMN.** The assays contained 450 μM Y17W msPapA, 1.9 μM prereduced PapB, 2 mM DTT, and 2.4 mM SAM. The assays with electron mediators contained 25 μM of either oxidized FMN (*green square*) or FldA (*red triangle*). Each experiment was carried out in triplicate and all replicates are shown. For mass spectra of sample withdrawn at 2, 4, 6, and 18 h, see Fig. S5 for prereduced PapB, Fig. S6 for prereduced PapB + FldA(ox), and Fig. S7 for prereduced PapB + FMN(ox).

The reaction catalyzed by PapB should be redox neutral. Once the RS cluster is reduced, PapB reductively cleaves SAM to generate dAdo⋅, which in turn initiates the chemistry by H-atom transfer from the substrate. Upon formation of the thioether cross-link, which oxidizes the C3 of Asp, the reducing equivalent is returned to the enzyme, presumably to AC1 based on the previously published selenocysteine (SeCys) peptide substitution that demonstrated direct binding and subsequent disengagement from an AC during selenoether formation (24). Therefore, one would expect that the enzymatic system would need to be primed once by prereduction and that subsequent turnover cycles would not require additional external reducing equivalents. To assess this, we reduced PapB in the presence of 10 mM NaDT after purification and reconstitution. The excess NaDT was removed by gel filtration, and the protein was concentrated before further chromatography using a high-resolution gel filtration column. A sample of the flow-through from the concentration step was kept as a control for experiments described later. In the experiments described below, a msPapA variant containing Y17W substitution was employed to permit a more accurate quantitation of the peptide stock. Fig. S6 shows the mass spectrum of an assay utilizing the Y17W msPapA after incubation with prereduced PapB. The data show that prereduced PapB is marginally catalytically active, as evidenced by the conversion of ∼3% of substrate to the cross-linked product in 2 h. Additionally, there is a time-dependent increase in the cross-linked product over the 18-h incubation (Figs. 5 and S6). The notable observation here is that, while the prereduced protein is catalytically active, the activity is not as robust as when the protein is assayed in the presence of excess reductant. Previously, we reported the estimated turnover number of PapB as 7.4 ± 0.1 s^–1^ when assayed in the presence of NaDT and 2.6 ± 0.2 s^–1^ when reduced by FldA/FPR/NADPH, when acting on the Y17W peptide as the substrate. While the activity with the prereduced enzyme is lower, we suspect that the reduction is not quantitative and only a small fraction of the PapB is reduced with NaDT in the prereduction conditions.

In the experiments shown in Figure 3, oxidized flavin (in solution or bound as in FldA/FPR) stimulates the activity of PapB. To determine the source of the stimulation, assays with the prereduced PapB were carried out in the presence of either *oxidized* FldA or FMN. Remarkably, the reaction rate is twice as fast in the case of FldA and more than 3 times as fast with FMN compared with prereduced PapB alone (see Fig. 5 for percent turnover for each respective scenario, and see Figs. S7 and S8 for MS spectra for each timepoint for the FldA and FMN scenarios, respectively). While the reaction remains slow with the prereduced enzyme, we observed complete conversion of the msPapA to the modified form in 18 h in the presence of either FldA or FMN. This is consistent with observations in Figure 3*B*, which show that inclusion of FMN alone leads to measurable, albeit slow, activity.

Our working mechanism for the activity of PapB is that the formation of the cross-linked product generates a singly reduced PapB, where the electron presumably resides in AC1. Therefore, to ready the system for a second round of catalysis, the electron must be cycled back to the RS cluster. The mechanism by which this electron is returned to the RS cluster is unknown. It has been proposed that this transfer is intramolecular. The results described above with assays containing FldA and FMN suggested to us that the process may be intermolecular. That is, for the electron to cycle back to the RS cluster, it must first be transferred from the protein to an external recipient.

### Postcatalysis reactivation of the RS cluster requires intermolecular electron transfer

**Figure 6:**
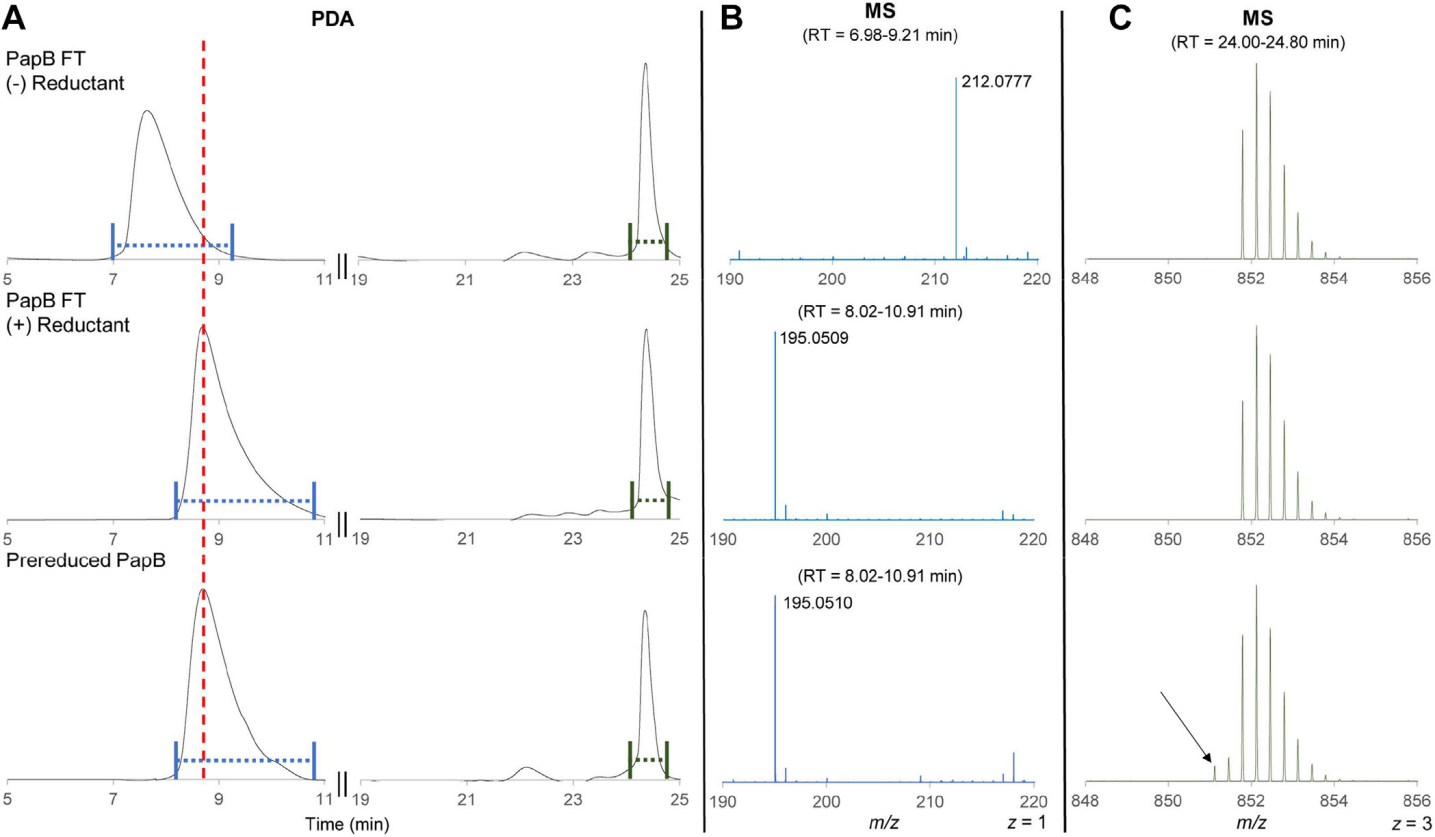
**Activation of QueE with prereduced PapB.***A*, Photodiode array (PDA)-detected chromatograms of reaction mixtures containing QueE, CPH_4_, and msPapA. Control reactions lacking any source of reducing equivalents show CPH_4_ and msPapA, eluting at ∼7.9 and ∼24 min, respectively. A sample of the flow-through (50 μl) from the PapB concentration step was added to control for the presence of any excess reductant. The identities of (*B*) CPH_4_ or CDG and (*C*) msPapA are confirmed by mass spectral analysis of the corresponding species in the mass spectrometric analyzer. CPH_4_ exhibits an *m/z* of 212.077 (expected monoisotopic mass = 212.0778, ppm error = −0.47). The absence of any CDG indicates no reducing equivalents sufficient for activation of QueE are present in the assay. As a positive control, the same experiment was carried out in the presence of NaDT (middle row). Unlike in the negative control shown in (*A*), the presence of NaDT leads to complete conversion of CPH_4_ to CDG, which elutes at 8.9 min and exhibits an *m/z* of 195.0510 (expected monoisotopic mass = 195.0513, ppm error = −1.54). As expected, since no PapB is present, no cross-links are observed in msPapA. In the presence of prereduced PapB, complete conversion of CPH_4_ to CDG is observed. CDG has the same retention time and *m/z* as the product made in the NaDT control (*middle column*, *middle row versus middle column*, *bottom row*). In addition, cross-linking of msPapA is observed, as evidenced by a 2 Da shift during the 18-h assay. The *arrow* in (*C*) shows the position of the monoisotopic peak of the cross-linked msPapB. Experimental conditions shown here are a representative spectrum of the t = 18 h timepoint. All of the assays contained 200 μM Y17W msPapA, 12 μM oxidized QueE, 2.43 mM CPH_4_, 2 mM MgSO_4_, 2 mM DTT, and 2.4 mM SAM. Additionally, the (+) Reductant assay (*second row*) contained 2 mM NaDT and the prereduced PapB assay (*third row*) contained 1.9 μM prereduced PapB and no NaDT.

To obtain evidence that intermolecular electron cycling is possible, at least in principle, prereduced PapB was incubated in the presence of another RS enzyme, 6-carboxy-6-deazaguanine synthase (QueE, Uniprot O31677), which, as purified, is catalytically inactive. QueE is a class 1 rSAM enzyme and catalyzes the conversion of 6-carboxy-5,6,7,8-tetrahydropterin (CPH_4_) to CDG (see Fig. S9) in the biosynthetic pathway to deazapurine-containing natural products (48, 49). QueE has been extensively characterized and contains a single 4Fe-4S cluster, which binds and upon reduction to the +1 state activates the SAM for reductive cleavage to form dAdo⋅. The dAdo⋅ initiates the catalytic cycle by H-atom transfer from the substrate (30, 35, 48, 49, 50, 51, 52). The reaction catalyzed by QueE is catalytic in SAM, as the cofactor is reformed upon formation of CDG (48). Prereduced PapB was assessed for its ability to activate oxidized as-isolated QueE. To ensure that the prereduced PapB sample did not have any residual reductant, the flow-through of the final concentration step (see above) was added to an assay mixture containing both CPH_4_ and msPapA substrates, as well as all the other assay components except for a reducing equivalent (Fig. 6, top row). An aliquot of this reaction mixture was analyzed by HPLC interfaced to both an MS and a UV-visible detector. The UV-visible and MS spectra of the species eluting at 7.9 min are consistent with CPH_4_, and there is no evidence for CDG in either chromatogram. Additionally, msPapA is unmodified, consistent with the modification requiring PapB, which is not present in the assay. Next, as a positive control, an identical reaction was set up containing 2 mM NaDT (Fig. 6, middle row). In this sample, the CPH_4_ is converted to CDG, as evidenced by the appearance of a new peak at 8.9 min with a UV-visible feature at 300 nm, as well as a species with *m/z* of ∼195.05 in the MS trace (Fig. 6, middle row, blue MS). As in the reaction containing the flow-through, there is no modification to msPapA because no PapB is present (Fig. 6, middle row, green MS). In the identical assay containing PapB (Fig. 6, bottom row), a peak in the HPLC traces consistent with CDG exhibiting a peak at 300 nm in the UV-visible trace and a *m/z* of 195.05 in the corresponding MS are observed. In contrast to the controls above, in the presence of prereduced PapB, msPapA is modified as well, evidenced by the 2 Da shift in the peptide MS envelope (Fig. 6, bottom row, green MS). Generally, these experiments show in principle PapB can transfer reducing equivalents to an unrelated electron acceptor (QueE), providing the first unambiguous evidence for intermolecular electron transfer between two unrelated rSAM proteins.

Additionally, the intermolecular activation experiment was carried out in reverse direction, where prereduced QueE was mixed with oxidized PapB under the same reaction conditions above. When the concentrations of PapB and QueE were held constant, no cross-linked msPapA was observed after 18 h (Fig. S10*A*). However, when the ratio of QueE:PapB was 10:1, approximately 50% of the Y17W msPapA was cross-linked after 18 h (Fig. S10*B*). This differential ability of QueE to pass reducing equivalents to PapB likely reflects the difference in the midpoint potentials of the proteins, which potentially favors localization on QueE. The reciprocal activation of QueE and PapB suggests that these rSAM enzymes are capable of exchanging electrons. Taken together with the activation of PapB with FMN/NADPH, or MiaB with ferredoxin-NADP^+^ oxidoreductase (36), it is plausible that, *in vivo*, redox active enzymes could activate rSAM enzymes. These observations also provide an explanation for the stimulatory effect of oxidized FldA or FMN observed in Figures 2 and 3, as either one can play a role in shuttling the reducing equivalent post catalysis to the RS cluster.

### Role of [4Fe-4S] clusters of PapB in intermolecular electron transfer

Based on sequence alignments (Fig. S2), predicted structures (Fig. 1*A*), and iron content, PapB can house three [4Fe-4S] clusters, any of which may be capable of reducing QueE. We do not know where the electron introduced during the prereduction resides in the protein, and therefore we could not determine which of the three clusters may be involved in the electron transfer to QueE. Therefore, three site-directed variants of PapB were generated: ΔRS, ΔAC1, and ΔAC2. Cys-to-Ala variants were constructed by site-directed mutagenesis, which removed all the putative coordinating Cys residues for each variant. The Cys to Ala mutations are as follows: ΔRS (C119, C123, and C126); ΔAC1 (C352, C370, and C421); and ΔAC2 (C408, C411, C417, and C440). Unlike WT PapB, which is highly stable and is readily obtained in pure form, these variants are substantially less stable (Fig. S11). We encountered significant difficulties while removing the purification tag after TEV cleavage, as the PapB variants became unstable and precipitated during elution from the HisTrap, which was used to separate the TEV-cleaved MBP from PapB. Therefore, the MBP tags were not cleaved in the subsequent studies with the mutants. SDS-PAGE analysis of the variants shows three major species: the higher-molecular-weight band (∼100 kDa) corresponds to the full-length MBP-PapB, and the lower-molecular-weight bands suggest significant cleavage of the MBP from PapB yielding the isolated tag alone (∼42 kDa) and PapB (∼54 kDa). ICP-MS analysis of the samples and comparison with the protein concentration from the Bradford assays indicates that the protein variants contain 2 ± 0.8 equivalents of Fe per mol of polypeptide, which suggests only a partial reconstitution had occurred.

The ΔRS, ΔAC1, or ΔAC2 variants were assessed for catalytic activity. When incubated with SAM under reducing conditions, the ΔRS variant did not produce any dAdo, whereas both the ΔAC1 and ΔAC2 variants did (Fig. S12) (53). Interestingly, while the MS spectra of the msPapA peptide revealed no cross-link with the ΔRS or ΔAC1 variants, the AC2 variant produces a small but measurable quantity of cross-linked product. The dAdo formation being reliant on the RS cluster is consistent with all established data with rSAM enzymes. To our knowledge, this is the first demonstration of an AC2 knockout that still produces the anticipated cross-linked product.

While the AC2 variant appears to be catalytically active, the enzyme only carries out a single turnover. Based on the ratio of modified to unmodified peptide in the MS, the same quantity of cross-linked product was formed in the reactions containing 1:1, 2:1, or 4:1 ratio of msPapA:ΔAC2 PapB after an 18-h incubation (Fig. S13). In each reaction no more than 10 μM of the msPapA is turned over. Since the concentration of ΔAC2 PapB was 40 μM in each assay, these data suggest that approximately one-fourth of the protein is fully reconstituted. Furthermore, the observation that the protein produces the same quantity of cross-linked product regardless of concentration of msPapB suggests that the stoichiometry of the reaction is limited by the PapB variant.

**Figure 7:**
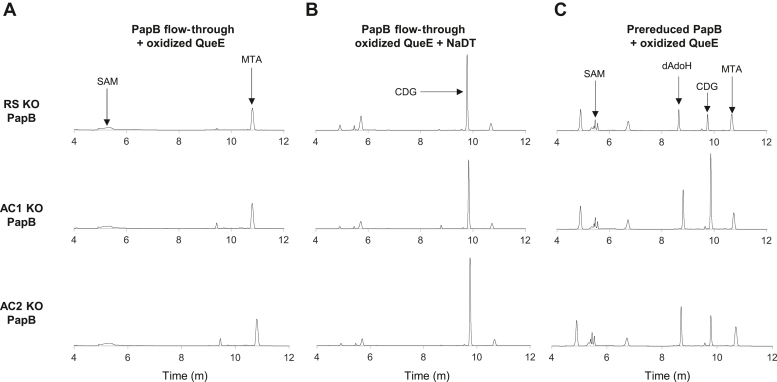
**Intermolecular electron transfer between prereduced PapB Fe-S cluster variants and oxidized QueE.** An aliquot of each assay containing oxidized QueE and (*A*) flow-through from the PapB variant, (*B*) NaDT, or (*C*) prereduced PapB was analyzed by LC-MS with in-line UV-visible (at 299 nm) and mass spectrometric detection. In all the cases, a small amount of methylthioadenosine is observed resulting from nonenzymatic hydrolysis of SAM. In the presence of NaDT, CDG is observed, consistent with the ability of NaDT to fully activate QueE. In the presence of prereduced PapB, CDG is formed in all samples. However, the amount of CDG produced is reduced in the ΔRS and ΔAC2 samples relative to the ΔAC1 samples. The comparison of the mass spectra between the observed and expected monoisotopic masses and ppm error for each peak in a prereduced WT PapB + oxidized QueE chromatogram are shown in Fig.S13. See Table S2 for a comparison of the relative CDG formed in each KO variant relative to the WT.

Next, the site-directed PapB variants were prereduced as described for the WT enzyme and assessed for their ability to support turnover of QueE. We hypothesized that the location of electron transfer between PapB and QueE could be determined by assaying ability of cluster mutants of PapB to reduce QueE, as measured by conversion of CPH_4_ to CDG. Figure 7 shows that, when QueE is incubated with 520 μM CPH_4_ in the presence of the flow-through from the corresponding prereduced PapB variant preparations, only methylthioadenosine (MTA) forms. This was expected as MTA is a common spontaneous degradation product of SAM, formed by nucleophilic attack of the carboxylate at C4 of SAM to eliminate MTA and form homoserine lactone (54). In a positive control, a peak for CDG eluting at 9.8 min is clearly visible when 2 mM NaDT is added to reduce QueE. When the flow-through is replaced with the prereduced PapB variants (ΔRS, ΔAC1, or ΔAC2) as the source of the reducing equivalent, CDG is present in each sample. However, the amount of CDG formed varies measurably. The formation of CDG is most efficient with the protein that still contains AC1, whereas the removal of AC2 or RS clusters reduces the amount of CDG formed by 50 to 70% (Fig. S14 and Table S2). These data suggest that, during the intermolecular activation of QueE, RS and AC2 are preferred. It is important to note at this point that there are no mediators (FldA, FPR, or FMN) in these reactions, so the electron transfer likely involves direct protein–protein interactions. In each variant, two clusters could potentially be present and the extent to which RS and/or AC2 are involved in the transfer remains unknown. We attempted to purify a variant where both the RS and AC2 were deleted. However, this protein variant was unstable and precipitated during the purification and could not be isolated.

## Discussion

The use of the dithionite reductant in rSAM enzymes can be traced back to assays conducted by Barker and coworkers in 1970 with lysine 2,3-aminomutase (55), 2 decades before the role of SAM in catalysis had been established by Frey and colleagues in 1987 (56). Within 5 years of the observations by Frey, Reichard and colleagues reported that FldA can reduce the rSAM enzyme ribonucleotide reductase (31). These studies provide the two key precedents that have led to the widespread use of either dithionite or FldA/FPR to reductively activate rSAM enzymes. In recent years, the FldA/FPR system has often been referred to as the biological/physiological reducing system, implying a role for these proteins (or their homologs) in reductive activation of rSAM enzymes *in vivo.*

The results presented here originated as follow-up experiments after we observed that PapB, which is active when assayed with FldA/FPR/NADPH, retained activity even when FldA, FPR, or NADH was absent (Fig. 2). Control follow-up experiments revealed that FldA can, in fact, be reduced by NADPH directly. We considered that the hypothesis of both FldA and FPR may be unnecessary for electron transfer from NADPH to the RS cluster and that the cofactor of FldA (FMN) may be the lynchpin for successful activation. Consistent with this hypothesis, we demonstrated that the FldA/FPR/NADPH system could be simplified to just NADPH and FMN (Fig. 3). Our findings suggest that the FldA/FPR pair, often regarded as the prototypical biological/physiological reducing system of rSAM enzymes, may not be essential for generalized enzyme activation. Instead, their prevalence as reducing systems may be largely due to their early adoption as the first flavin-containing reducing system investigated with rSAM enzymes. The fact that FMN, upon reduction with NADPH, can support activity supports the notion that the reductive activation simply requires a redox-active species that can deliver reducing equivalents in one-electron steps. This explains how the *E. coli* reducing pair of FldA and FPR can support many rSAM enzymes from different kingdoms of life. In this scenario, the reduction process is clearly agnostic to the scaffold that binds the reducing system. We strongly suggest that the field consider FMN/NADPH as an alternative to the harsher NaDT or the more cumbersome FldA/FPR/NADPH systems to reductively activate rSAM enzymes.

Many rSAM enzymes that harbor multiple iron–sulfur clusters, in principle, require only an initial one-electron priming step to carry out multiple turnovers (42). Studies on PapB have shown that, upon prereduction, the protein is able to carry out multiple rounds of catalysis. However, we were surprised that the reaction with prereduced PapB was slow relative to reactions containing NaDT. Two explanations for the lower efficiency were considered. First, it is likely that only a small fraction of the protein is reduced, leading to less activity compared with the NaDT-containing reactions. Second, after a single round of turnover, the electron deposited in AC1 may not be efficiently transferred back to the RS cluster without an intermolecular redox shuttle. The latter hypothesis led to experiments showing that oxidized FldA or FMN accelerated the rate of the reaction, thereby providing the first indication for possible intermolecular electron transfer in PapB.

Further experiments were carried out with mixtures of QueE and PapB to investigate the intermolecular electron transfer hypothesis more directly. Purified QueE is catalytically inactive and requires reductive activation. Remarkably, prereduced PapB activates QueE. Using ΔRS, ΔAC1, and ΔAC2 variants of PapB we can show that activation of QueE requires either the RS or AC2 to be present. Removal of AC1 does not lead to a change in the levels of active QueE, whereas removal of RS or AC2 substantially alters the level of QueE activity. Additionally, we show that ΔAC2 PapB is itself catalytically active, albeit in a single turnover. These results suggest that the reduction of AC2 is an obligatory way station for the electron on the pathway from a postcatalysis active site state (electron in AC1) to the reductively activated state (electron in RS).

**Figure 8:**
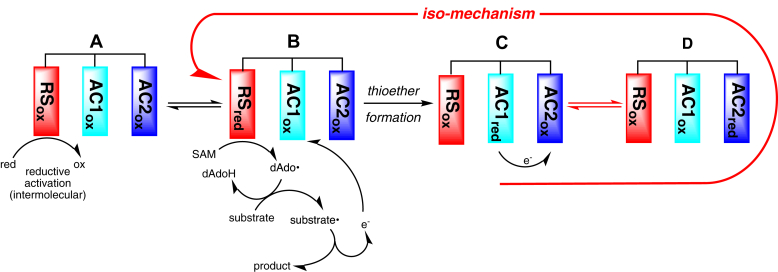
**Redox states and cycling in PapB.** When purified, PapB (*A*) is fully oxidized. Upon reductive activation of the RS cluster to (*B*), the RS cluster catalyzes the cleavage of SAM to dAdo⋅, which initiates the chemistry by H-atom transfer from the substrate. A reducing equivalent is then transferred to AC1 concomitant with thioether bond formation to generate the postcatalysis state (*C*). Isomerization of the enzyme to the catalytically active RS reduced state (*B*) requires an intramolecular electron transfer from AC1 to AC2 forming (*D*), followed by an intermolecular transfer involving a redox active species to form the reduced RS state (*B*). The iso-mechanism involves conversion of (*C*) to (*B*) via (*D*). The enzyme only requires a single priming reductive activation step, with all subsequent cycles relying on the electronic isomerization to reactivate the protein.

A working model that incorporates these observations is shown in Figure 8. The as isolated form of the enzyme is depicted as one where the RS cluster is oxidized (state A). Reductive activation of the enzyme to form state B entails reduction of the RS cluster. Binding of substrate to AC1 is followed by reductive cleavage of SAM, leading to H-atom transfer from the substrate to dAdo⋅ to generate a substrate-based radical (Fig. 1, *B* and *C*). This intermediate undergoes cross-linking with the Cys thiolate with concomitant transfer of an electron into AC1 forming state C in Figure 8. At this point, the protein is one electron reduced, but the electron does not reside in the RS cluster, where it must be for reductive cleavage of SAM in the subsequent catalytic cycle. Our data suggest that the reduction of the RS cluster requires an intermolecular transfer of the electron ejected into the AC1 out of the protein, likely through AC2, with its subsequent transfer to the RS cluster. The experiments with ΔAC2 support this critical role for AC2 in the intermolecular electron transfer (state D in Fig. 8). We note that the initial activation of the RS cluster in the as-purified protein could proceed by direct reduction of the RS cluster. Also, in solution, PapB molecules could be exchanging electrons, such that the AC2 cluster of one protein can reduce the RS cluster of another protein. This could explain why PapB is active with NaDT in the absence of any obvious oxidants to shuttle the electron from the AC2 to the RS cluster. In addition to providing mechanistic insights into the function of RS enzymes, this model could be used to guide future studies on the reductive activation of members of the rSAM superfamily.

Presumably PapB and other rSAM enzymes with multiple clusters, where the catalytic cycle concludes with a reducing equivalent residing in the protein, must undergo a redox isomerization step that returns the reducing equivalent back to the RS cluster. This is a special case of the iso-mechanism that has long been considered a possibility in many enzyme-catalyzed reactions (57). In an iso-mechanism, the catalytic cycle leaves the enzyme in a form that is not capable of undergoing another round of reaction before an isomerization step involving the free enzyme occurs. This type of a mechanism is commonly invoked in reactions that, for example, require isomerization to shift protonation states of the acid/base catalyst in racemases (57). In Figure 8, the iso steps are depicted by red arrows. In PapB and related systems, the isomerization that moves the electron from the AC cluster to the RS cluster is complex, requiring not only an intramolecular electron transfer from AC1 to AC2 but also a subsequent isomerization that transfers the electron to an external acceptor, followed by transfer back to the RS. This process is depicted in red arrows within the dashed area of Figure 8.

The implications of these results are significant and likely generalizable to the entire superfamily of rSAM enzymes. From a practical perspective, the FMN/NADPH system is potentially a general method for reductive activation of rSAM enzymes. Unlike the strong reductant, NaDT, the FMN/NADPH reactivity is limited by the midpoint potential of the flavin, which is significantly more positive than NaDT (58, 59) and, therefore, less likely to lead to side reactions. However, perhaps the most significant aspect of this work is instead the first demonstration of intermolecular electron exchange between completely unrelated rSAM enzymes. This shows that, in principle, *any* redox active protein that can deliver a single electron can partner with an rSAM enzyme, including other rSAM enzymes. This leads to a model for *in vivo* activation that suggests a link between activity and the redox state of the cell.

The reductive activation of rSAM enzymes and the role of AC clusters are two enigmas in the rSAM field. The role of AC1 clusters in rSAM enzymes has come into sharper focus over the last few years, with discoveries that suggest it plays a key role in catalysis (19, 20, 21, 24). The present study begins to shed light on the role of AC2, which when present, is often relegated to generic electron transfer steps. We are mindful that PapB is a member of the SPASM family of rSAM enzymes and that several rSAM enzymes that catalyze similar transformations also belong to the Twitch group and do not have an AC2. For example, SkfB, which catalyzes the cross-linking of a Cys thiolate to the α-carbon of an amino acid in SkfA, only has an RS and an AC1. It remains to be seen if the intermolecular model is generalizable. The results presented here provide a framework in which to explore roles of ACs and provide a new model to direct future studies of the reductive activation of redox enzymes.

## Experimental procedures

### Expression of PapB and PapB variants (PapB∗)

PapB or PapB∗ was cotransformed with pPH151 (*suf* operon) (60, 61), grown and induced with IPTG, and harvested after 16 h, as described (40).

### Purification of PapB and prereduced PapB

All processes involving PapB were carried out in a Coy anaerobic chamber (97% N_2_/3% H_2_). PapB was purified as described (40). To prepare the prereduced protein, immediately following reconstitution and desalting of excess Fe and S ions, 10 mM sodium dithionite (NaDT) was added to the protein mixture and stirred at room temperature for 10 min. The NaDT was removed by a BioGel P6 DG (100–200 mesh) desalting column, which had been equilibrated into 0.05 M Pipes⋅NaOH (pH 7.4) buffer containing 20% glycerol, 2 mM DTT, and 0.3 M KCl. The eluate was concentrated to ∼4 ml in an Amicon concentrator (YM-10 membrane) under 40 psi N_2_. An additional gel filtration step employing Cytiva XK26 (1000 mm) S-column equilibrated with 0.05 M Pipes⋅NaOH (pH 7.4) containing 10% glycerol, 2 mM DTT, and 0.3 M KCl was employed, with the protein eluting at 2.6 ml/min isocratically. The fractions containing pure PapB were identified by UV trace, dark brown color, and Coomassie-stained SDS-PAGE gel. The pooled fractions were concentrated to ∼1 ml, flash frozen in liquid N_2_, and stored at −80 °C.

### Construction of each PapB Cys→Ala variant

Each of the cluster Cys deletion variants of PapB was constructed using site-directed mutagenesis with nonoverlapping primers (Table S1). Standard protocol for PCR with Phusion DNA polymerase (NEB #M0530) was used to generate modified plasmids. Briefly, after PCR amplification using appropriate primers, the template DNA was digested with *Dpn*I (NEB #R0176L) for 1 h at 37 °C to remove template DNA. An aliquot (10 μl) of the *Dpn*I-treated sample was added to a mixture of 2 μl T4 DNA ligase buffer (NEB # B0202S), 1 μl of T4 DNA ligase (NEB #M0202S), 1 μl of T4 polynucleotide kinase (NEB #M0201S), and 6 μl of H_2_O. The mixture was incubated at 20 °C for 2 h to phosphorylate and ligate the new plasmid. The sequence of each construct was verified by sequencing at the University of Utah Core facilities. For each cluster deletion variant, all the putative coordinating Cys residues were mutated to Ala in the final constructs (RS: C119, C123, and C126; AC1: C352, C370 and C421; and AC2: C408, C411, C417, and C440).

### Purification of prereduced [4Fe-4S] deletion variants of PapB

Prereduced cluster deletion MBP-PapB was prepared as follows: after initial elution from the HisTrap HP columns (GE healthcare), the fractions containing MBP-PapB∗ were identified by light brown color and SDS-PAGE analysis. The pooled fractions were then desalted into a buffer containing 0.05 M Pipes⋅NaOH (pH 7.4), 2 mM DTT, 0.3 M NaCl, and 15% glycerol (v/v). The concentration of protein in the sample was determined by the Bradford method utilizing bovine serum albumin as a standard. The variants of MBP-PapB were reconstituted by mixing 10 molar equivalents of 0.1 M Na_2_S and 0.1 M FeNO_3_. The Fe was added first in 5-μl aliquots, followed by the 5-μl Na_2_S aliquots, allowing 10 s between each addition to ensure thorough mixing. The resulting reconstitution mixture was stirred for 3 h at room temperature, after which it was centrifuged for 10 min at 16,000*g* to pellet any precipitated enzyme. The protein sample was desalted into buffer containing 0.05 M Pipes⋅NaOH (pH 7.4), 0.3 M NaCl, 2 mM DTT, and 15% glycerol (v/v) to remove any excess FeNO_3_ or Na_2_S *via* BioGel P6 DG desalting gel 100-200 mesh (wet) (Bio-Rad). Sodium dithionite (NaDT) was added to the protein mixture to 10 mM final concentration, and the 40 ml solution was stirred at room temperature for 10 min followed by another desalting step to remove excess NaDT into the same buffer conditions. The eluate was then concentrated to a minimal volume (∼2 ml) in an Amicon concentrator (YM-10 membrane) under N_2_ at 40 psi. Following the concentration, 100-μl aliquots of each variant were resuspended in 400 μl of fresh desalting buffer and reconcentrated in a 10 kDa MWCO centrifugal concentrator (Millipore # UFC5010). This step was repeated 3 times to ensure all excess reductant was removed. Any residual NaDT that would have been left over after the desalting step would have been negligible with the nearly >5000-fold dilution.

### Purification of FldA

FldA was expressed and purified as described (30) with the following modifications. Each step was carried out in a Coy anaerobic chamber (97% N_2_/3% H_2_). After sonication and pelleting of the cellular debris for 45 min at 18,448*g*, the cell lysate was loaded onto a DEAE Sepharose ion-exchange column (2.6 x 12.5 cm) that had been equilibrated in 20 mM Tris⋅HCl (pH 7.2) buffer containing 10 mM DTT and 1 mM EDTA. FldA was eluted with a 500 ml linear gradient to buffer containing 20 mM Tris⋅HCl (pH 7.2), 10 mM DTT, and 0.5 M KCl. Fractions containing pure FldA were identified by UV trace, orange color, and Coomassie-stained SDS-PAGE gel. The pooled fractions were dialyzed twice into 4 L of 20 mM Hepes (pH 7.4), 150 mM KCl, and 2 mM DTT and then concentrated to ∼1.5 ml, flash frozen in liquid N_2_, and stored at −80 °C.

### Purification of FPR

FPR was expressed and purified as described (30).

### Purification of QueE, prereduced QueE, and CPH_4_

QueE and CPH_4_ were expressed and purified as described (51). Prereduced QueE was generated using the same reconstitution method as described for PapB.

### Preparation of minimal substrate PapA (msPapA)

msPapA was prepared by solid phase peptide synthesis using either a Prelude peptide synthesizer (Protein Technologies Inc) or a Chorus peptide synthesizer (Protein Technologies Inc). The syntheses and subsequent purification followed the same procedure as reported (40). All of the Fmoc-amino acids were purchased from Protein Technologies Inc. The msPapA was quantified by dry weight and the Y17W msPapA was quantified by its absorbance at 280 nm.

### Assays with reconstituted PapB

The assays were carried out in a Coy Laboratories anaerobic chamber (97% N_2_/3% H_2_) at room temperature. All of the reactions contained 0.05 M Pipes⋅NaOH (pH 7.4), 2 mM DTT, and 2 mM SAM. SAM was synthesized enzymatically from ATP and methionine and purified as described (62). msPapA (∼200 μM), 15% glycerol, 300 mM KCl, and 1.9 to 6 μM standard (*i.e.*, no prereduction) PapB was used for each reaction. When used, the biological reducing system contained 25 μM FldA, 2 μM FPR, and 2 mM NADPH. Scenarios that utilized the biological reducing system cofactors instead of the respective proteins contained 25 μM FMN and 2 mM NADPH. All of the assays were quenched by the addition of 10% of the reaction volume with 30% (w/v) trichloroacetic acid. The samples were centrifuged at 16,000*g* for 10 min to pellet any precipitated proteins.

### Assays with prereduced PapB

The assays were carried out in a Coy Laboratories anaerobic chamber with 97% N_2_/3% H_2_ atmosphere at room temperature. All of the reactions contained 0.05 M Pipes⋅NaOH (pH 7.4), 2 mM DTT, 2.4 mM SAM, 200 to 450 μM Y19W msPapA, 15% glycerol, 300 mM KCl, and 1.9 μM prereduced PapB. The assays that included redox mediators contained either 25 μM FMN or 25 μM FldA. The assays that utilized QueE contained the following: 12 μM QueE, 520 μM to 2.43 mM CPH_4_, and 2 mM MgSO_4_. All of the assays were quenched by the addition of 10% of the reaction volume with 30% (w/v) trichloroacetic acid. The samples were centrifuged at 16,000*g* for 10 min to pellet any precipitated proteins.

### U/HPLC-MS analysis of reactions

All of the assays and controls were analyzed as described (30, 40) with the following modifications for the assays that contained both prereduced wildtype (WT) PapB and QueE. A 50-μl aliquot was injected onto a Hypersil GOLD C18 column (2.1 mm x 150 mm, 1.9 mm particle size) (Thermo Fisher) preequilibrated in 0.1% (v/v) Optima TFA (Fisher) in LC-MS Optima water (Fisher). The separation consisted of washing with 100% A (0.1% [v/v] TFA in Optima water) from 0 to 3 min, followed by a linear gradient from 0 to 40% B (0.1% [v/v] TFA in Optima acetonitrile) from 3 to 20 min, followed by a linear gradient from 40% B to 100% B from 20 to 23 min, washing with 100% B from 23 to 26.5 min, and re-equilibration into 100% A from 26.5 to 30.1 min.

The assays that contained a PapB variant and QueE were analyzed as follows. A 20-μl aliquot was injected onto a Hypersil GOLD C18 column (2.1 mm x 150 mm, 1.9 mm particle size) (Thermo Fisher) preequilibrated in 0.1% (v/v) Optima TFA (Fisher) in LC-MS Optima water (Fisher). The separation consisted of washing with 100% A (0.1% [v/v] TFA in Optima water) from 0 to 3 min, followed by a linear gradient from 0 to 20% B (0.1% [v/v] TFA in Optima acetonitrile) from 3 to 20 min, followed by a linear gradient from 20% B to 100% B from 20 to 23 min, washing with 100% B from 23 to 26.5 min, and re-equilibration into 100% A from 26.5 to 30.1 min.

## Data availability

All data and results are contained within the main text and supplemental information.

## Supporting information

This article contains supporting information (34, 46, 63).

## Conflict of interest

V. B. and K. A. S. E. have disclosed the results to the University of Utah, which holds patent interests in the findings.
