# Supplementary material for: Intermolecular electron transfer in radical SAM enzymes as a new paradigm for reductive activation
Source: J Biol Chem. 2023 Jul 17;299(9):105058. doi: 10.1016/j.jbc.2023.105058 (PMC10470005; doi:10.1016/j.jbc.2023.105058)
Supplement: Supporting information [file mmc1.pdf]

Intermolecular electron transfer in radical SAM enzymes as a new paradigm for reductive activation

Karsten A. S. Eastman, Andrew S. Jochimsen, Vahe Bandarian*

University of Utah, Department of Chemistry, 315 S 1400 E, Salt Lake City, Utah 84112

*Vahe Bandarian

Email: vahe@chem.utah.edu

Table of Contents

Table S1. Primers for site-directed mutagenesis of PapB to produce cluster KO variants. Page S-3

Figure S1. SDS-PAGE analysis of reconstituted and purified PapB on a 12% crosslinked gel. Page S-4

Figure S2. Sequence alignment with PapB, CteB and anSME. Page S-5

Figure S3. Representative mass spectra of assays containing msPapA with PapB, SAM, FldA and FPR. Page S-6

Figure S4. Simulation of mass spectral envelopes cross-linking isotopic envelopes for unmodified msPapA, modified msPapA, and mixtures of the two. Page S-7

Figure S5. Assessing reduction of FldA by DTT. Page S-8

Figure S6. Y17W msPapA is crosslinked by pre-reduced PapB. Page S-9

Figure S7. Y17W msPapA is efficiently crosslinked by pre-reduced PapB in the presence of oxidized FldA. Page S-10

Figure S8. Y17W msPapA is efficiently crosslinked by pre-reduced PapB in the presence of oxidized FMN. Page S-11

Figure S9. QueE formation of CDG from CPH_4. Page S-12

Figure S10. Activation of PapB in the presence of pre-reduced QueE. Page S-13

Figure S11. SDS-PAGE of the cluster deletion variants of PapB. Page S-14

Figure S12. ΔAC1 and ΔAC2 variants of PapB reductively cleave SAM to form dAdoH. Page S-15

Figure S13. ΔAC2 PapB produces crosslinked msPapA. Page S-16

Figure S14. Activation of QueE with pre-reduced PapB. Page S-17

Table S2. Pre-reduced PapB KO variant peak intensities relative to the WT assay Page S-18

Table S1. Primers for site-directed mutagenesis of PapB to produce cluster KO variants.

Residue Mutation	Forward Primer	Reverse Primer
RS cluster: C119A	gcgAACCTTAGATGTACTTACTGTTATGG	TTCTTGCACCATAAATAAGGTCAG
RS cluster: C123A	gcgACTTACTGTTATGGGG	TCTAAGGTTCGCTTCTTGC
RS cluster: C126A	gcgTATGGGGAGGAAGG	GTAAGTCGCTCTAAGGTTCG
AC1: C352A	gcgGGAGCGGGTAC	AAAGTGGATTCTAGGACCGC
AC1: C370A	gcgCATCGTTTTGTAGGC	AGGGAACAAATTTCCCCTAACATC
AC1: C421A	gcgCATCAAGAGAACTTCG	TCCACCACCACAAAGATTTTTG
AC2: C408A	gcgTCAAAGTGTTGGGC	GGTGGTTCTATTTCTTACTGTTGAATTC
AC2: C411A	gcgTGGGCCAAAAATCTTTG	CTTTGACGCGGTGGTTC
AC2: C417A	gcgGGTGGTGGATGC	AAGATTTTTGGCCCACGC
AC2: C440A	gcgAAAGTAACTAAGAATTTCATTAACG	AAGTTTACCGACAGGTTGATTG

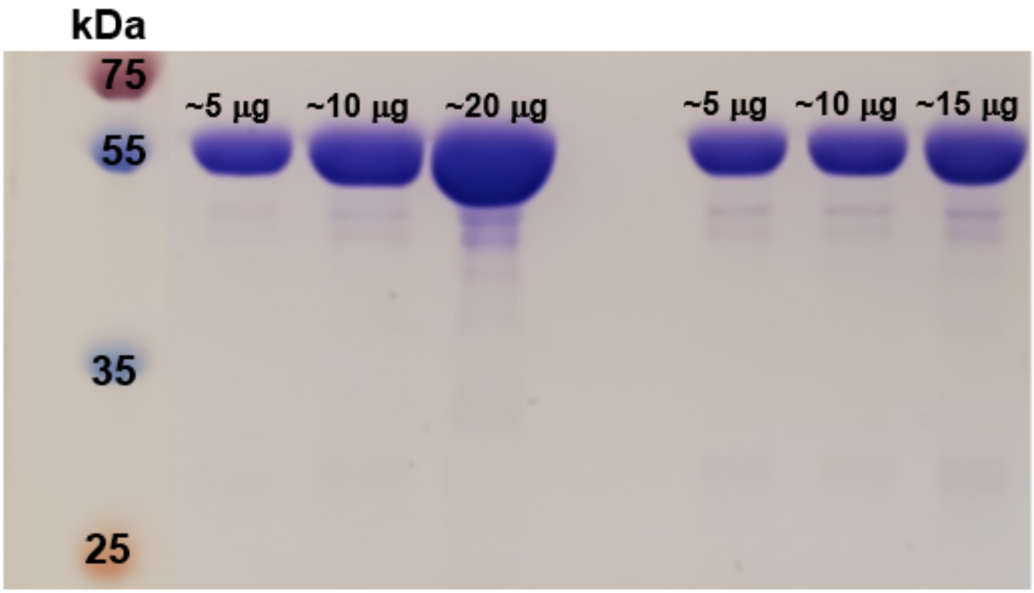

Fig. S1. SDS-PAGE analysis of reconstituted and purified PapB on a 12% crosslinked gel. Lanes 2, 3 and 4 show standard (i.e., no pre-reduction) PapB. Lanes 6, 7 and 8 show pre-reduced PapB.

```
PapB:    …QECNLRCTYCYGEEGEYNQKG---KMTSEIARSAVDFLIQQSGEIEQLNITFFGGEPLL
CteB:    …HDCNLRCKYCFASTGNFGGQR---NMMSLEVGKKAIDFLISESGNRKNLEIDFFGGEPMM
AnSME:   …SGCNLKCTYCFYHSLSDNRNVKSYGIMRDEVLESMVKRVLNEA--NGHCSFAFQGGEPTL

PapB:     NF-PLIQETVQYVHEQSEIHNKKFSFSITTNGTLITPKIKNFFYKHHFAVQTSIDGDEKT
CteB:     NF-DVVKGIIEYARQKEKEHNKNFRFTLTTNGLLLNDENIKYINENMQNIVLSIDGRKEV
AnSME:    AGLEFFEKLMEL-QRKHNYKNLKIYNSLQTNGTLIDESWAKFLSENKFLVGLSMDGPKEI

PapB:     HNFNRFFKGGQGSYDLLLKRTE---EMRNDRKIGARGTVTPAELDLSKSF---DHLVKLG
CteB:     NDRMRIRIDGSGCYDDILPKFKYVAESRNQDNYYVRGTFTRENMDFSNDV---LHLADEG
AnSME:    HNLNRKDCCGLDTFSKVERAAELFKKYKVEFNIL--CVVTSNTARHVNKVYKYFKEKDFK

PapB:     FRK--IYLSPALYSLSDDHYDTLSKEMVKLVEQFRELLEREDYVTAKKMS-NVL-GMLS-
CteB:     FRQ--ISVEPVVAA-KDSGYDLREEDLPRLFEEYEKLAY--EYVKRRKEG-NWF-NFFHF
AnSME:    FLQFINCLDPLYEEKGKYNYSLKPKDYTKFLKNLFDFWY-EDFLNGNRVSIRYFDGLLET

PapB:     -KIHSGG----PRIHFCG---AGTNAAAVDVRGNLFPCHRFVGEDECSIGNLFDEDPL--
CteB:     MIDLTQGPCIVKRLTGCG---SGHEYLAVTPEGDIYPCHQFVGNEKFKMGNVKEGVLN--
AnSME:    --I------LLGKSSSCGMNGTCTCQFVVESDGSVYPCDFYV-LDKWRLGNIQDMTMKEL

PapB:     --SKQYNFIENSTVRNRTTCSKCWAKNLCGGGCHQENFAENGNVNQPVGKLCKVTKNFIN…
CteB:     --RDIQNYFKNSNVYTKKECDSCWAKFYCSGGCAANSYNFHKDINTVYKVGCELEKKRVE…
AnSME:    FETNKNHEFIKLSFKVHEECKKCKWFRLCKGGCRRCRDSKE-DSALELNYYCQSYKEFFE…
```

Fig. S2. Sequence alignment with PapB, CteB and anSME. The alignment was generated with Clustal Omega.(63) The residues that coordinate to the RS cluster are colored red, AC1 is colored yellow, and AC2 is colored purple. Unlike anSME, which has four Cys residues coordinated to AC1(34), PapB appears to be similar to CteB(46) and based on sequence alignment and predicted structure—only contains three coordinating Cys residues on AC1.

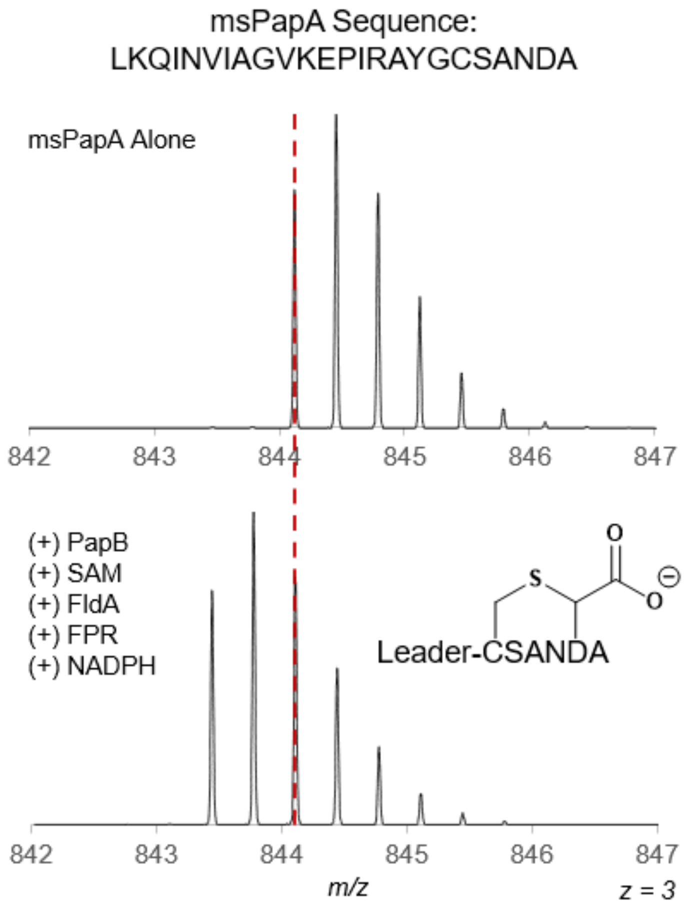

Fig. S3. Representative mass spectra of assays containing msPapA with PapB, SAM, FldA and FPR. The msPapA peptide in the presence of 1.9 μM PapB, 2 mM SAM, 2 mM DTT, 25 μM FldA, 2 μM FPR and 2 mM NADPH forms a thioether between a Cys thiol and Cb of Asp in CX_3D motifs, as evidenced by a 2 Da mass shift between the unmodified peptide sequence (expected monoisotopic mass: 844.1197; observed monoisotopic mass: 844.1198; ppm error: 0.19; *z* = 3) and modified peptide sequence (expected monoisotopic mass: 843.4478; observed monoisotopic mass: 843.4480; ppm error: 0.24; *z* = 3) is visible by high resolution mass spectrometry. The enzyme buffer contained 0.05 M PIPES•NaOH (pH 7.4), 300 mM KCl, 2 mM DTT, and 15% glycerol.

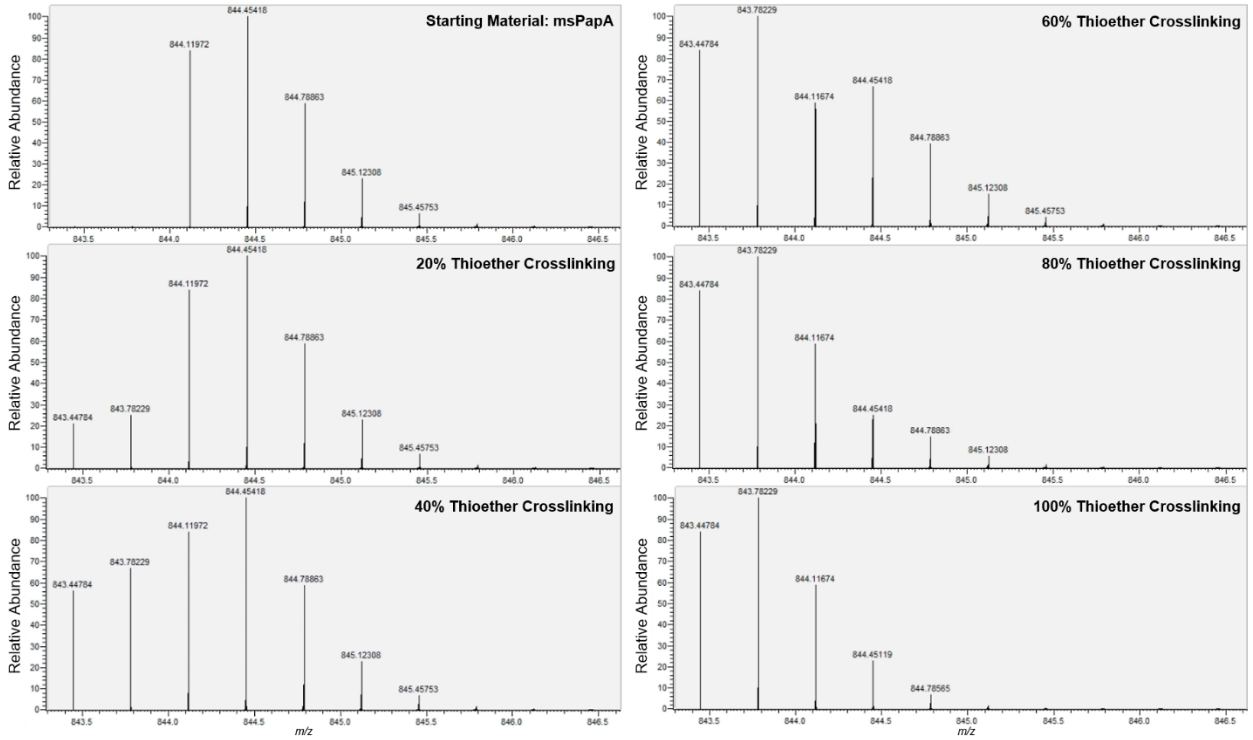

Fig. S4. Simulation of mass spectral envelopes cross-linking isotopic envelopes for unmodified msPapA, modified msPapA, and mixtures of the two. The peptide isotopic envelopes were simulated using Thermo Xcalibur Qual Browser version 4.1.31.9. The starting material formula (C110H184N32O34S) was mixed with the thioether crosslink formula (C110H182N32O34S) using a H-atom adduct and a charge distribution of 3. Spectra were computed for 0, 20, 40, 60, 80, and 100% conversion of msPapA to the thioether species.

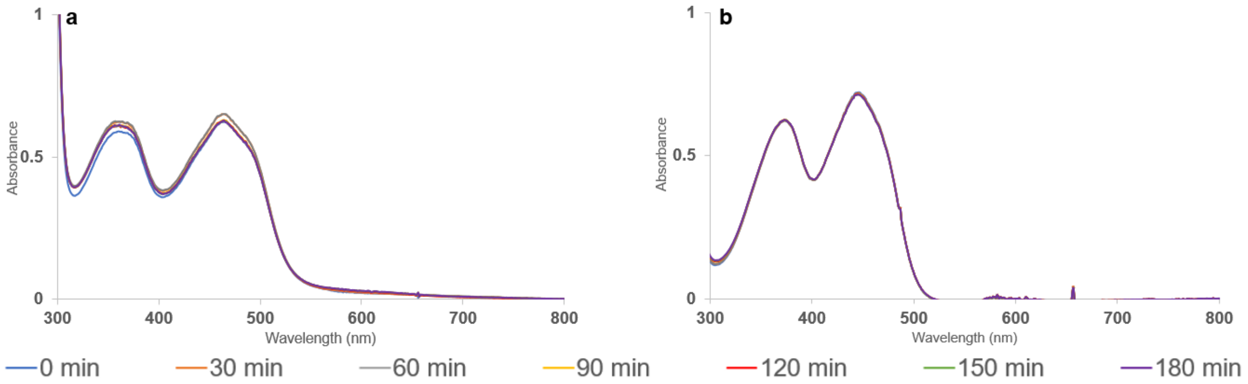

Fig. S5. Assessing reduction of FldA by DTT. Each experiment was conducted in triplicate with representative spectra from one of the runs shown. In a buffer of 50 mM PIPES•NaOH (pH 7.4), 300 mM KCl and 2 mM DTT, no reduction of either FldA (a) or FMN (b) is seen over 180 min.

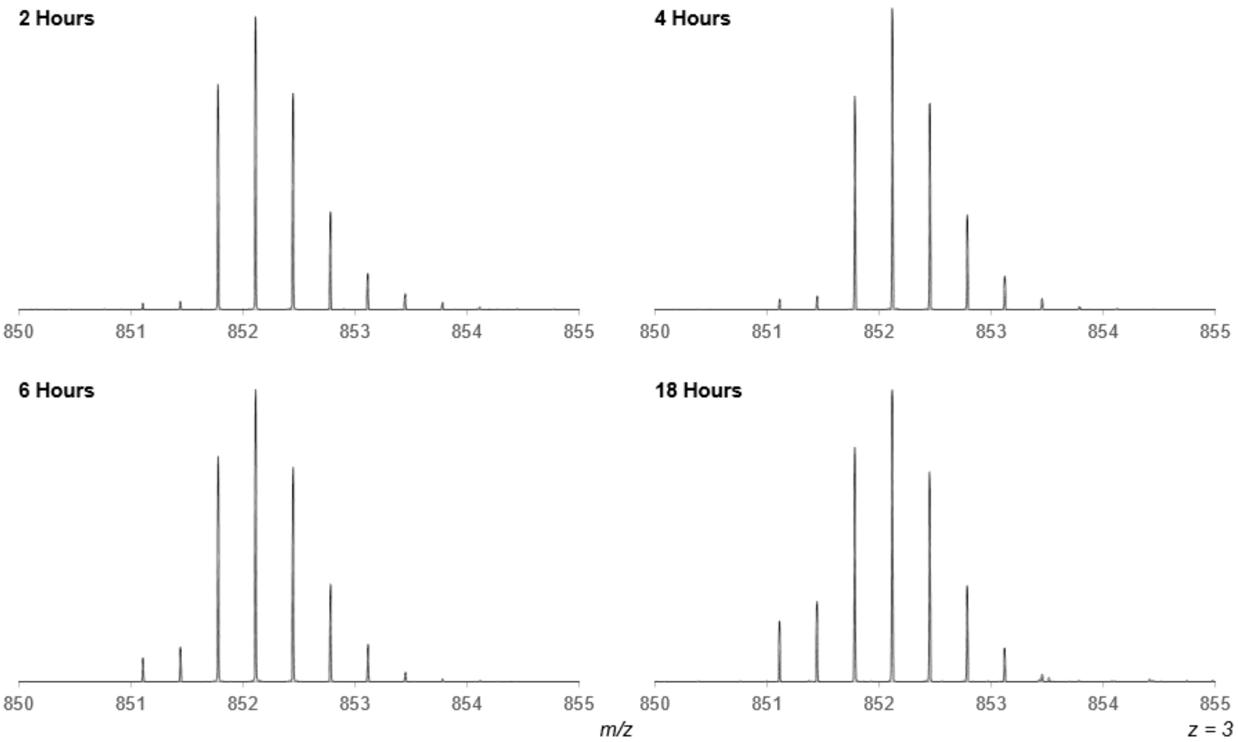

Fig. S6. Y17W msPapA is crosslinked by pre-reduced PapB. Assays contained 1.9 µM pre-reduced PapB, 450 µM Y17W msPapA, 2.4 mM SAM, and 2 mM DTT. Samples were withdrawn at 2, 4, 6 and 18 h and analyzed for crosslinking. Assays were run in triplicate with a representative spectrum for each timepoint shown.

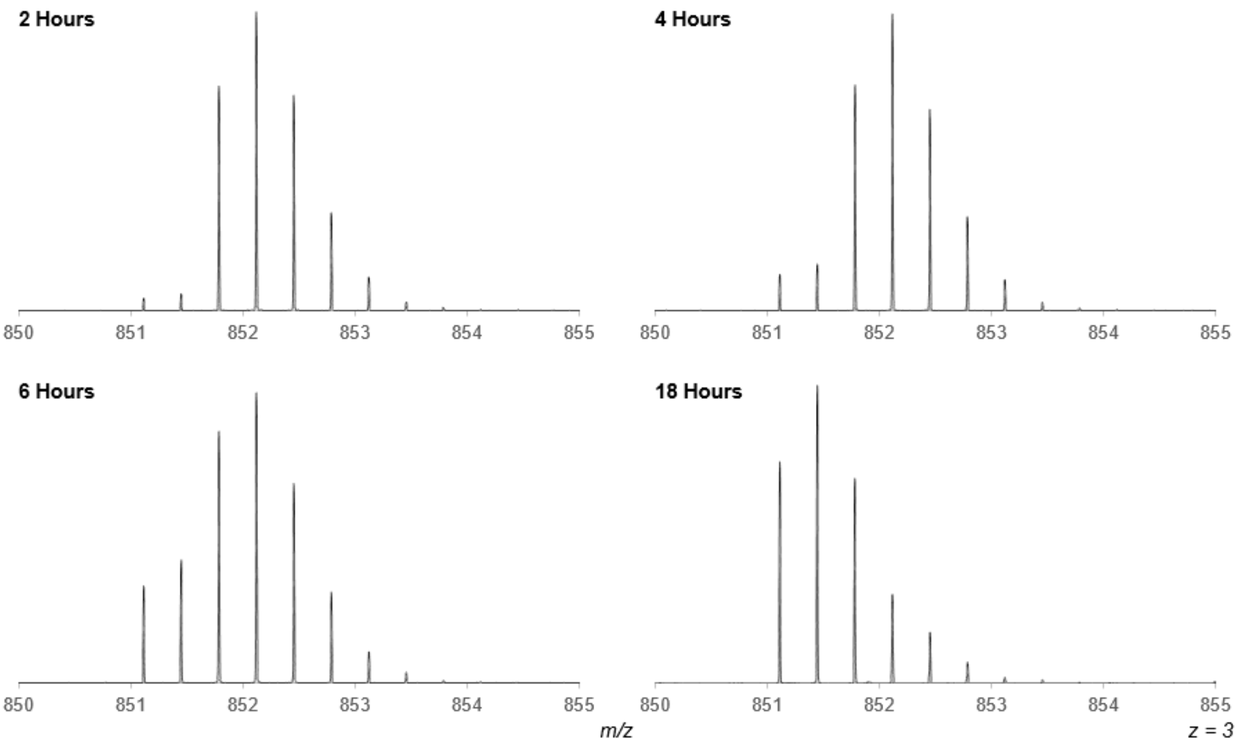

Fig. S7. Y17W msPapA is efficiently crosslinked by pre-reduced PapB in the presence of oxidized FldA. Assays contained 1.9 μM pre-reduced PapB, 450 μM Y17W msPapA, 25 μM FldA, 2.4 mM SAM, and 2 mM DTT. Samples were withdrawn at 2, 4, 6 and 18 h and analyzed for crosslinking. Assays were run in triplicate with a representative spectrum for each timepoint shown.

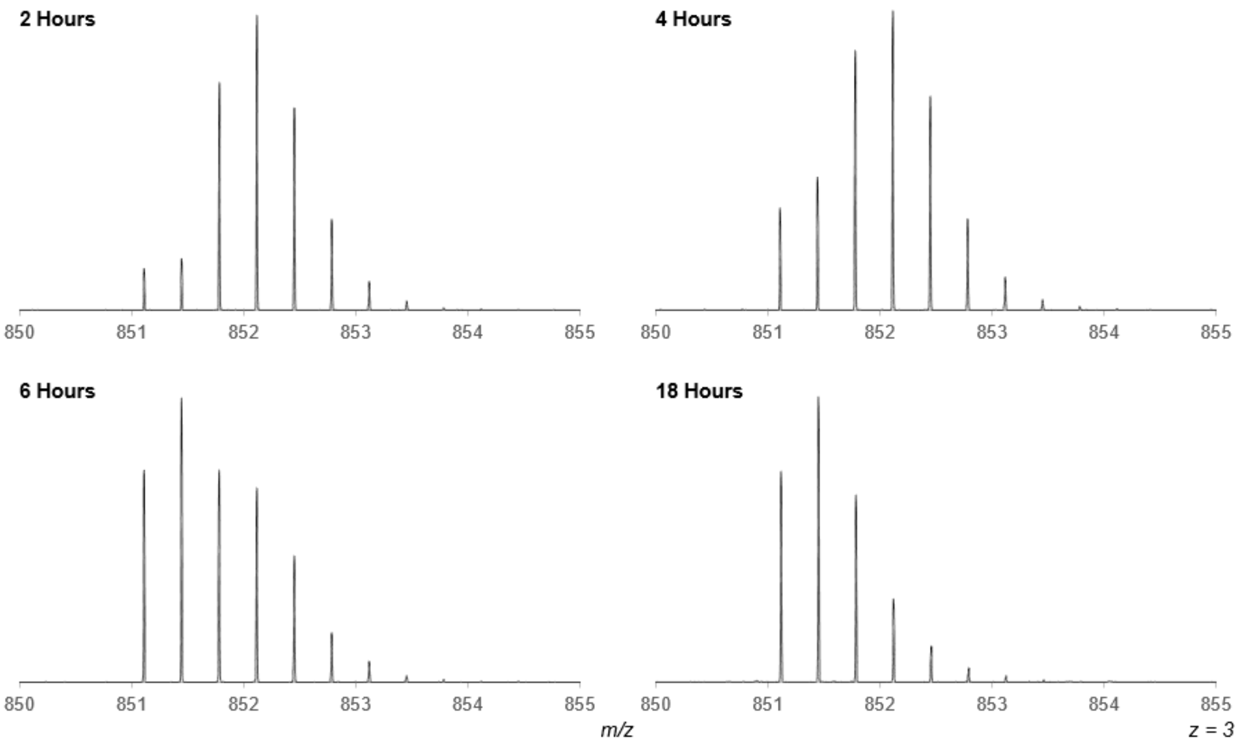

Fig. S8. Y17W msPapA is efficiently crosslinked by pre-reduced PapB in the presence of oxidized FMN. Assays contained 1.9 μM pre-reduced PapB, 450 μM Y17W msPapA, 25 μM oxidized FMN, 2.4 mM SAM, and 2 mM DTT. Samples were withdrawn at 2, 4, 6 and 18 h and analyzed for crosslinking. Assays were run in triplicate with a representative spectrum for each timepoint shown.

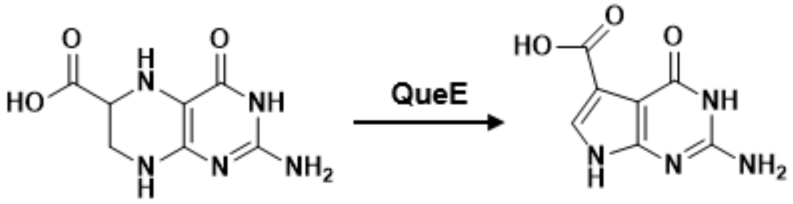

Fig. S9. QueE formation of CDG from CPH_4. QueE generates CDG from the radial-mediated ring contraction of CPH_4.

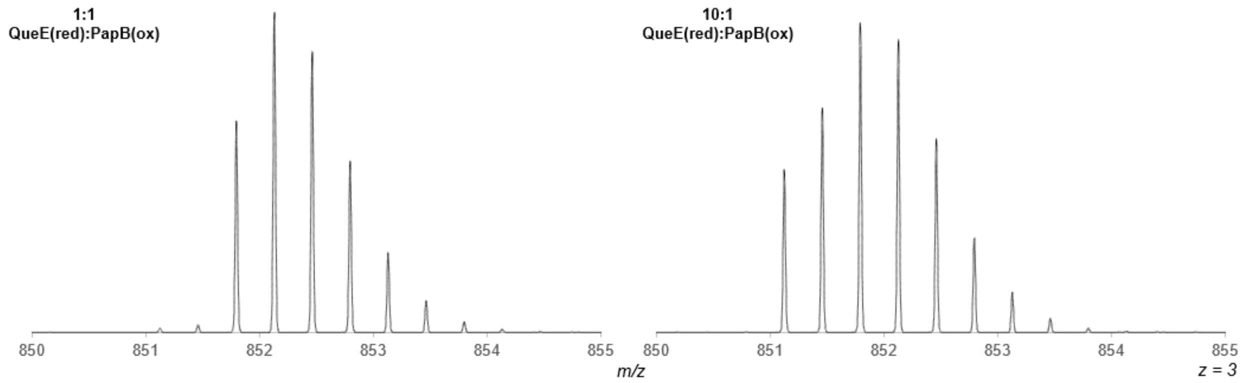

Fig. S10. Activation of PapB in the presence of pre-reduced QueE. The assays contained 450 μM Y17W msPapA, 2.4 mM SAM, 2 mM DTT, 2 mM $MgSO_4$, and variable QueE(red) and PapB(ox). When the enzyme concentrations are in a 1:1 ratio (5 μM:5 μM), only trace amounts of crosslinked Y18W msPapA are observed after 18 h (left). In the presence of a 10:1 ratio of pre-reduced QueE to PapB (50 μM:5 μM), approximately 50% of msPapA is crosslinked after 18 h (right).

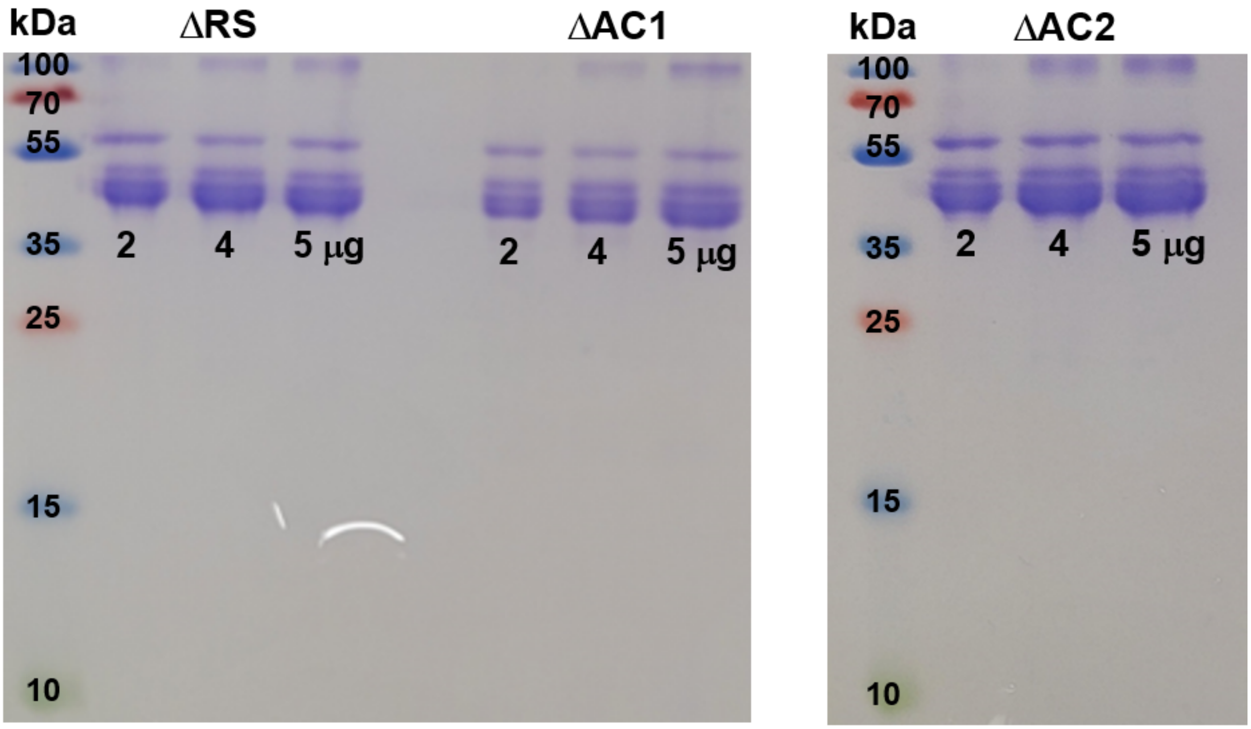

Fig. S11. SDS-PAGE of the cluster deletion variants of PapB. Lanes 2, 3 and 4 show the ΔRS variant, lanes 6, 7 and 8 show the ΔAC1 variant. On a second gel, lanes 2, 3 and 4 show the ΔAC2 variant. In each case, a minimal amount of the MBP-PapB fusion is seen at the 100 kDa mark. The band at the 55 kDa mark is PapB. The band between 35 and 55 kDa are proteolyzed MBP.

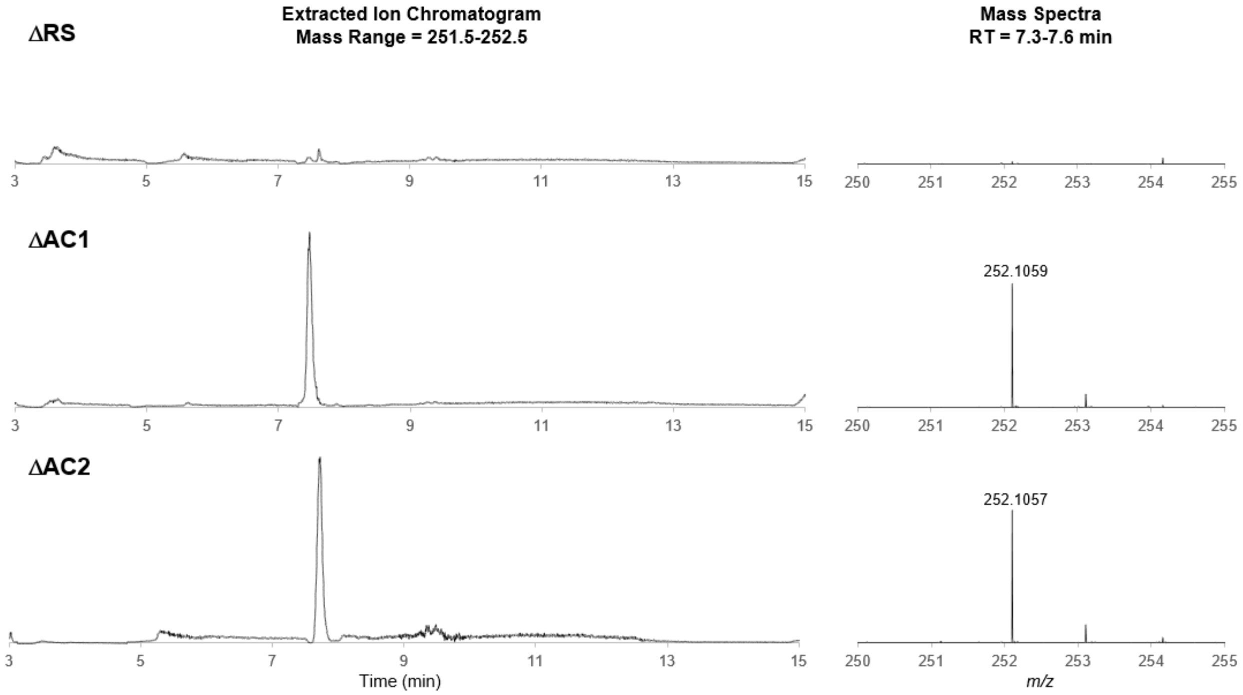

Fig. S12. ΔAC1 and ΔAC2 variants of PapB reductively cleave SAM to form dAdoH. PapB variants (5 μM) were incubated with 2.4 mM SAM and 2 mM NaDT over 18 h. An extracted ion chromatogram (EIC) of masses between *m/z* 251.5-252.5 is shown for each variant. The mass spectra for retention time (RT) range of 7.3-7.6 min are shown to the right of each EIC. For ΔAC1, the observed *m/z* is within 2.77 ppm of the expected monoisotopic mass for dAdoH (252.1052, *z* = 1). For ΔAC2, the observed mass within 1.99 ppm of the expected monoisotopic mass for dAdoH. The ΔRS variant does not form any detectable dAdoH.

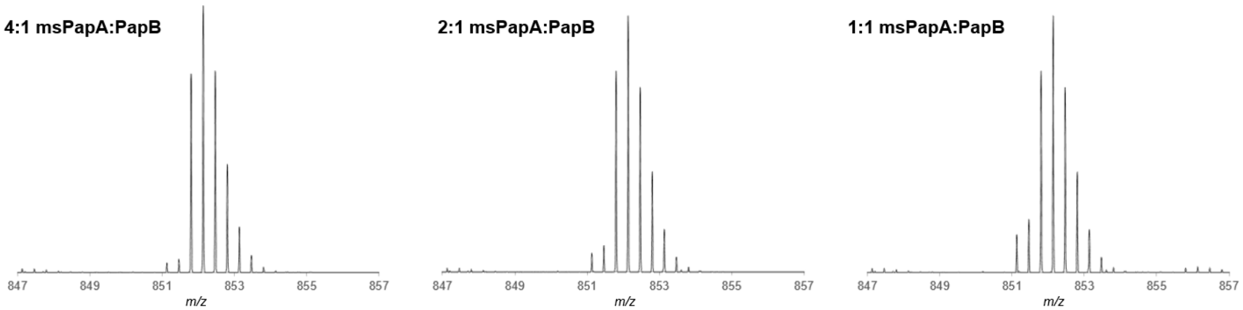

Fig. S13. ΔAC2 PapB produces crosslinked msPapA. PapB (40 μM) was incubated with either 160, 80, or 40 μM Y17W msPapA in the presence of 2.4 mM SAM, 2 mM DTT, 0.05 M PIPES•NaOH (pH 7.4), 300 mM KCl, and 15% glycerol for 18 h. The PapB concentration in each assay was held constant and the assay results indicate that no more than ~10 μM of msPapA turned over in each case.

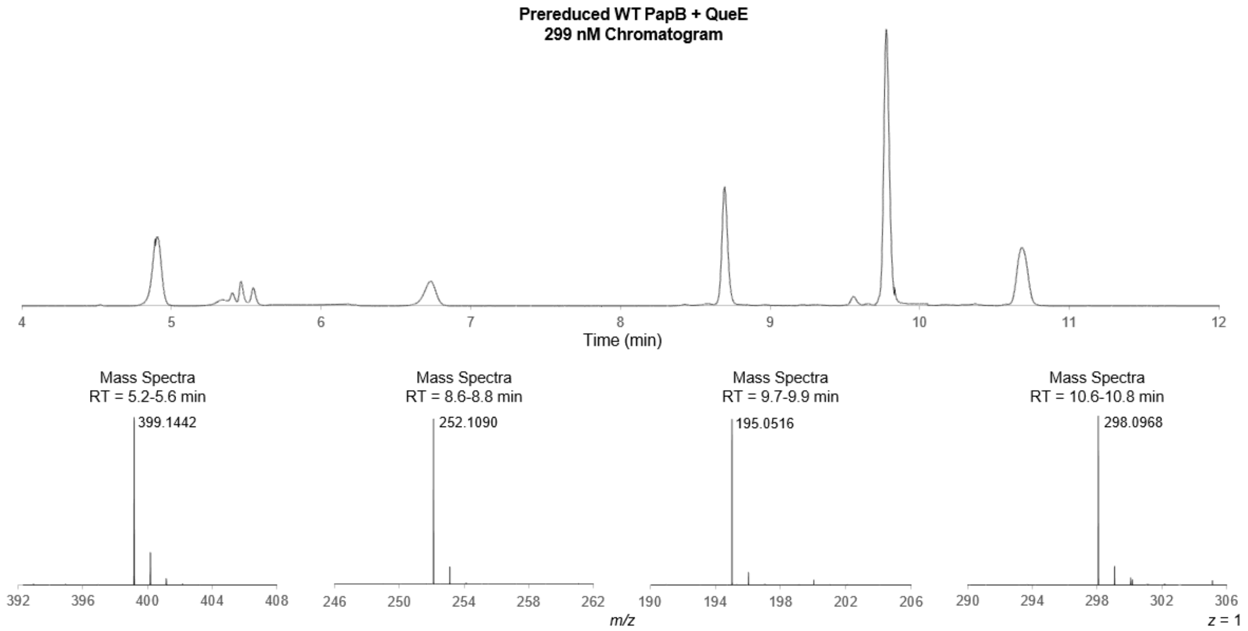

Fig. 14. Activation of QueE with pre-reduced PapB. Assay mixtures contained 2 mM DTT, 2 mM SAM, 10% glycerol, 300 mM KCl, 5 µM QueE, 0.5 mM CPH_4, and 2 mM $MgSO_4$ in 0.05 M PIPES•NaOH (pH 7.4). The chromatogram was obtained at 299 nm. The weak signals are observed for several peaks (such as CPH_4) in this spectrum are due to their lower extinction at 299 nm. Under these conditions, SAM elutes between 5.2-5.6 min (expected monoisotopic mass = 399.1445, observed monoisotopic mass = 399.1442, ppm error = -0.75; z = 1), dAdoH elutes between 8.6-8.6 min (expected monoisotopic mass = 252.1091, observed monoisotopic mass = 252.1090, ppm error = -0.40; z = 1), CDG elutes between 9.7-9.9 min (expected monoisotopic mass = 195.0513, observed monoisotopic mass = 195.0516, ppm error = 1.54), and MTA elutes between 10.6-10.8 min (expected monoisotopic mass = 298.0968, observed monoisotopic mass = 298.0968, ppm error = 0; z = 1).

Table S2. Pre-reduced PapB KO variant peak intensities relative to the WT assay. The ΔAC1 produces the smallest amount of change relative to the WT assay. Both the ΔRS and the ΔAC2 variants produce substantially less CDG when incubated with oxidized QueE.

CDG Peak intensity relative to WT	ΔRS PapB	ΔAC1 PapB	ΔAC2 PapB
Replicate 1	34%	89%	45%
Replicate 2	38%	82%	49%
Replicate 3	32%	79%	52%
